# Calcineurin Governs Thermotolerance and Virulence of *Cryptococcus gattii*

**DOI:** 10.1534/g3.112.004242

**Published:** 2013-03-01

**Authors:** Ying-Lien Chen, Virginia N. Lehman, Yonathan Lewit, Anna F. Averette, Joseph Heitman

**Affiliations:** * Department of Molecular Genetics and Microbiology, Duke University Medical Center, Durham, North Carolina 27710; † Department of Plant Pathology and Microbiology, National Taiwan University, 106 Taipei, Taiwan; ‡ Department of Biomedical Engineering, Duke University, Durham, North Carolina 27710

**Keywords:** temperature sensitivity, pathogenicity, fluconazole tolerance, FK506, cyclosporin A, cation homeostasis, melanin, capsule

## Abstract

The pathogenic yeast *Cryptococcus gattii*, which is causing an outbreak in the Pacific Northwest region of North America, causes life-threatening pulmonary infections and meningoencephalitis in healthy individuals, unlike *Cryptococcus neoformans*, which commonly infects immunocompromised patients. In addition to a greater predilection for *C. gattii* to infect healthy hosts, the *C. gattii* genome sequence project revealed extensive chromosomal rearrangements compared with *C. neoformans*, showing genomic differences between the two *Cryptococcus* species. We investigated the roles of *C. gattii* calcineurin in three molecular types: VGIIa (R265), VGIIb (R272), and VGI (WM276). We found that calcineurin exhibits a differential requirement for growth on solid medium at 37°, as calcineurin mutants generated from R265 were more thermotolerant than mutants from R272 and WM276. We demonstrated that tolerance to calcineurin inhibitors (FK506, CsA) at 37° is linked with the VGIIa molecular type. The calcineurin mutants from the R272 background showed the most extensive growth and morphological defects (multivesicle and larger ring-like cells), as well as increased fluconazole susceptibility. Our cellular architecture examination showed that *C. gattii* and *C. neoformans* calcineurin mutants exhibit plasma membrane disruptions. Calcineurin in the *C. gattii* VGII molecular type plays a greater role in controlling cation homeostasis compared with that in *C. gattii* VGI and *C. neoformans* H99. Importantly, we demonstrate that *C. gattii* calcineurin is essential for virulence in a murine inhalation model, supporting *C. gattii* calcineurin as an attractive antifungal drug target.

*Cryptococcus gattii* and *Cryptococcus neoformans* are closely related basidiomycetous yeast species that cause the disease cryptococcosis after inhalation by the host. Cryptococcosis can cause pneumonia and disseminates throughout the body to infect other tissues, especially the central nervous system, causing meningoencephalitis (Perfect and Casadevall 2012). Cryptococcal meningoencephalitis is 100% fatal if untreated, and in certain human immunodeficiency (HIV)-infected populations, mortality rates of 100% have been reported within 2 wk after clinical presentation to health care facilities (French *et al.*, 2002). It is estimated that cryptococcosis causes more than 600,000 deaths per year (Park *et al.*, 2009). Unlike *C. neoformans*, which predominantly causes disease in immunosuppressed persons such as organ transplant recipients or HIV/acquired immunodeficiency syndrome (AIDS) patients, *C. gattii* frequently causes disease in both immunocompetent and immunosuppressed hosts (Chaturvedi and Chaturvedi 2011; Sorrell 2001). Although *C. neoformans* is distributed worldwide, it was previously thought that *C. gattii* was restricted to tropical regions of the world (Kwon-Chung and Bennett 1984). However, *C. gattii* emerged on Vancouver Island, Canada, in 1999, afflicting both residents and animals of the island. In 2006, the first case in the United States of infection by a *C. gattii* strain indistinguishable from the Vancouver Island VGIIa/major outbreak strain was identified in a patient from Orcas Island in Washington state (Upton *et al.* 2007; Datta *et al.* 2009). *C. gattii* has continued to spread throughout the Pacific Northwest and down the western coast and has emerged as a primary pathogen in the northwestern United States (Byrnes *et al.* 2010).

*C. gattii* is divided into four molecular types: VGI, VGII, VGIII, and VGIV (Bovers *et al.* 2008). Types VGI and VGII cause the majority of infections that occur in otherwise-healthy individuals, whereas VGIII and VGIV more commonly infect immunosuppressed HIV/AIDS patients (Byrnes *et al.* 2011). VGI is the most commonly isolated molecular type worldwide (Sorrell 2001), whereas the VGII molecular type is the cause of the Vancouver Island/Pacific Northwest outbreak (Fraser *et al.* 2005; Byrnes *et al.* 2010; Kidd *et al.* 2004). Type VGII can be further subdivided in VGIIa/major and VGIIb/minor, which were identified in the Vancouver Island outbreak (Kidd *et al.* 2004), as well as VGIIc/novel, which has emerged in Oregon and is causing illness in the region, along with isolates of the VGIIa and VGIIb genotypes (Byrnes *et al.* 2010).

Virulence studies have revealed both shared and unique molecular virulence attributes for *C. neoformans* and *C. gattii*. For example, the superoxide dismutase Sod1 is a prominent antioxidant that is required for virulence of *C. gattii* but not *C. neoformans* (Narasipura *et al.* 2003). Trehalose functions as an antioxidant and stress protectant and is produced by the enzyme trehalose-6-phosphate synthase, which is encoded by the *TPS1* and *TPS2* genes. Both Tps1 and Tps2 were found to be critical for thermotolerance, pathogenicity, and other virulence attributes (capsule and melanin production) in *C. gattii* (Ngamskulrungroj *et al.* 2009). However, the homologous genes in *C. neoformans* are required for thermotolerance but not for capsule or melanin production (Petzold *et al.* 2006). An interesting evolutionary switch of the cAMP-activated protein kinase A (Pka1 and Pka2) has been found in *C. gattii* and *C. neoformans*. Pkal governs mating, virulence, capsule, and melanin production in *C. neoformans* but only controls capsule production in *C. gattii* (D’Souza *et al.* 2001; Hicks and Heitman 2007). Pka2 does not regulate mating, capsule, or melanin production in *C. neoformans*, whereas the corresponding protein regulates these functions in *C. gattii* (D’Souza *et al.* 2001; Hicks and Heitman 2007). Ste12α is a transcription factor that regulates melanin, mating, virulence, and ecological fitness in *C. gattii* but only regulates mating and capsule size in *C. neoformans* (Ren *et al.* 2006; Yue *et al.* 1999). Finally, Gat1 is a GATA transcription factor required for virulence in *C. gattii* but not in *C. neoformans* (Ngamskulrungroj *et al.* 2012a). Meanwhile, *C. neoformans* Gat1 plays a greater role in the use of nitrogen sources such as glycine and creatinine compared with *C. gattii* Gat1 (Ngamskulrungroj *et al.* 2012a).

Calcineurin is a calcium-calmodulin activated serine-threonine specific protein phosphatase composed of a catalytic A (Cna1) and regulatory B calcium-binding subunit (Cnb1). Active calcineurin is an AB heterodimer, and loss of the B subunit can result in destabilization of the A subunit, causing calcineurin malfunction (Chen *et al.* 2010a). Pathogenic fungi require calcineurin for establishing infection via distinct mechanisms: (1) growth at body temperature (*C. neoformans* and *C. glabrata*) (Chen *et al.* 2012; Odom *et al.* 1997b); (2) survival in serum (*C. albicans*) (Blankenship *et al.* 2003); (3) filamentous growth (*A. fumigatus* and *C. dubliniensis*) (Chen *et al.* 2011; Steinbach *et al.* 2006); and (4) appressorial formation (*Magnaporthe oryzae*) (Choi *et al.* 2009). In this study we tested the pathogenic roles of calcineurin of *C. gattii* compared with *C. neoformans*, particularly with respect to the relevance to virulence and antifungal drug resistance of the *C. gattii* isolates causing the expanding Pacific Northwest outbreak.

## Materials and Methods

### Yeast strains, growth media, and chemicals

Yeast strains used in this study are listed in Table 1. The following media were used in this study: yeast extract peptone dextrose (YPD; 1% yeast extract, 2% peptone, 2% glucose) liquid medium and agar (2%) plates. YPD medium containing 100 µg/mL nourseothricin was used to select transformants. The following supplements were added to the media at the concentrations indicated: FK506 (Astellas Pharma Inc.), cyclosporin A (CsA; LC Laboratories), sodium dodecyl sulfate (Fisher), calcofluor white (fluorescent brightener 28) (Sigma-Aldrich), Congo red (Sigma-Aldrich), tunicamycin (Sigma-Aldrich), and fluconazole (Bedford Laboratories). Niger seed (*Guizotia abyssinica*) agar plates was made with 70 g of ground niger seed in 350 mL of distilled water (dH_2_O), filtered through cheese cloth with the addition of 1 g of dextrose, 20 g of Bacto agar, and dH_2_O added to 1 L. L-DOPA agar plates was made with 20 g of Bacto agar autoclaved in 900 mL of dH_2_O followed by addition of 1 g of L-asparagine, 1 g of dextrose, 3 g of KH_2_PO_4_, 0.25 g of MgSO_4_, 100 mg of L-DOPA, and 1 mg of thiamine. The pH was adjusted to 5.6, and dH_2_O was added to 1 L. Low iron media was made with 5 g of dextrose, 5 g of asparagine, 400 mg of K_2_HPO_4_, 80 mg of MgSO_4_, 250 mg of CaCl_2_, and 20 mg of ethylenediamine-*N*,*N*′-bis(2-hydroxyphenylacetic acid) followed by the adjustment of pH to 7.4 in 1 L of dH_2_O; then, 1 mL of 1000× vitamin-mineral mixture (400 mg of thiamine, 57 mg of boric acid, 5 mg of CuSO_4_, 10 mg of MnCl_2_, 2000 mg of ZnSO_4_, and 460 mg of sodium molybdate in 1 L of dH_2_O) was added after autoclaving.

**Table 1:** *Cryptococcus gattii* and *Cryptococcus neoformans* strains

Strain	Parent	Genotype	Source of Reference
*C. gattii*			
R265	Clinical isolate	Wild-type VGIIa MATα, serotype B	(Kidd *et al.* 2004)
YL136	R265	*cna1*Δ::*NAT*	This study
YL137	R265	*cna1*Δ::*NAT*	This study
R272	Clinical isolate	Wild-type VGIIb MATα, serotype B	(Kidd *et al.* 2004)
YL165	R272	*cna1*Δ::*NAT*	This study
YL169	R272	*cna1*Δ::*NAT*	This study
YC875	YL165	*cna1*Δ::*CNA1* (native locus)	This study
YC879	YL165	*cna1*Δ::*NAT CNA1* (ectopic)	This study
YC883	YL165	*cna1*Δ::*NAT* suppressor mutation	This study
YC886	YL165	*cna1*Δ::*NAT* suppressor mutation	This study
WM276	Environmental isolate	Wild-type VGI MATα, serotype B	(Kidd *et al.* 2005)
YL277	WM276	*cna1*Δ::*NAT*	This study
YL344	WM276	*cna1*Δ::*NAT*	This study
*C. neoformans*			
H99	Clinical isolate	Wild-type MATα, serotype A	(Perfect *et al.* 1993)
KK1	H99	*cna1*Δ::*NAT*	(Kojima *et al.* 2006)
KK5	KK1	*cna1*Δ::*NAT CNA1*::*NEO*	(Kojima *et al.* 2006)
JLCN146	H99	*lac1*Δ::*HYG*	(Nelson *et al.* 2003)

### Identification of *C. gattii* calcineurin gene orthologs

The *C. gattii* orthologs of the gene encoding the *C. neoformans* calcineurin catalytic subunit (Cna1) were identified by reciprocal BLAST searches between the two species, with the reciprocal best BLAST hit orthologs in *C. gattii* being *CNA1* (CNBG_5632 from R265; CGB_I3140C from WM276). *C. gattii* R265 Cna1 shares 99.4% and 97.0% identity over the full protein length with its corresponding *C. gattii* WM276 and *C. neoformans* H99 orthologs, respectively (Supporting Information, Figure S1A). In addition to the catalytic domain, *C. gattii* Cna1 has the conserved calcineurin B binding, calmodulin binding, and autoinhibitory domains (Cyert *et al.* 1991; Rusnak and Mertz 2000) (Figure S1).

### Disruption of the *C. gattii* calcineurin genes

All deletion strains were generated from the prototrophic *C. gattii* R265 (VGIIa), R272 (VGIIb), and WM276 (VGI) strains. The nourseothricin (*NAT*) resistance gene from plasmid pAI3 was used as a dominant selectable marker in a biolistic transformation protocol with a Bio-Rad model PDS-1000/He biolistic particle delivery system (Toffaletti *et al.* 1993). All primers used in strain construction are listed in Table S1. The *cna1*Δ mutant was generated in the *C. gattii* strains R265 (VGIIa), R272 (VGIIb), and WM276 (VGI) by overlap PCR as previously described (Davidson *et al.* 2002). For VGII strains, the 5′ and 3′ noncoding regions of the *CNA1* gene were amplified with primers JC174/JC175 and JC176/JC177 from R265 or R272 genomic DNA, and the dominant selectable marker (*NAT*) was amplified with the M13 universal primers (JC65/JC66) from plasmid pAI3. The *CNA1* gene replacement cassettes were generated by overlap PCR with flanking primers JC178/JC179, precipitated onto gold microcarrier beads (0.6 µm; Bio-Rad Laboratories, Inc., Hercules, CA), and the VGII strains R265 and R272 were biolistically transformed.

For the VGI strain WM276, the 5′ and 3′ regions of the *CNA1* gene were amplified with primers JC207/JC208 and JC209/JC210 from genomic DNA. The *CNA1* replacement cassette was generated by overlap PCR with primers JC211/JC212, and WM276 was biolistically transformed. Stable transformants were selected on YPD medium containing 100 µg/mL nourseothricin. Two independent nourseothricin-resistant *cna1* mutants (YL136 and YL137 from R265; YL165 and YL169 from R272; and YL277 and YL344 from WM276) derived from two separate transformations were obtained (Table 1). Mutants were confirmed by PCR and Southern blot analysis.

### Complementation of R272 calcineurin mutant and isolation of suppressor mutations at 38°

*C. gattii* R272 *cna1*Δ::*NAT* mutant YL165 was grown overnight in 50 mL of YPD medium containing 100 μg/mL nourseothricin, washed with dH_2_O, and resuspended in 12.5 mL of dH_2_O. Three-hundred microliters were spread to YPD medium containing 1 M sorbitol and incubated at 24° for 4 hr, and biolistic transformation was then performed as described previously. The 4.6-kb complementation DNA fragment used for biolistic transformation was amplified with primers JC174/JC177 from R272 genomic DNA and contained the 5′ noncoding region (NCR), *CNA1* gene, and 3′NCR. Because calcineurin is essential for growth at 37°, we screened transformants in YPD medium at a slightly more stringent condition of 38°. Transformants able to grow at 38° were colony purified and replica-plated to YPD medium containing 100 μg/mL nourseothricin. Both nourseothricin-sensitive and -resistant colonies were observed. The nouseothricin-sensitive colony YC875 (Table 1; Figure S7) represents native locus complementation from which the *NAT* selectable marker within the *cna1* mutant YL165 was replaced by wild-type *CNA1* gene (thus resulting in nourseothricin sensitivity), whereas nouseothricin resistant colonies represent ectopic *CNA1* integration (YC879; Table 1; Figure S7), or suppressor mutations (YC883 and YC886; two independently derived mutants; Table 1; Figure S7).

### Transmission electron microscopy

Cells were pelleted and rinsed in PBS buffer and resuspended in 3% glutaraldehyde in 0.1 M sodium cacodylate buffer (pH = 6.8) at room temperature for 1 hr. The cells were then transferred to 4° and incubated in fixative for several weeks. The cells were rinsed with three changes of cold 0.1 M sodium cacodylate buffer (pH = 6.8) by centrifugation and resuspended in fresh buffer and then postfixed with 2% OsO_4_ in the same buffer for 2 hr on ice in the dark. Cells were again rinsed with three changes of buffer as described previously. After the last buffer wash, the cells were centrifuged and the pellet was embedded in warm (60°) 2% agarose prepared in the same buffer. The tubes were spun for 5 min while the agarose was warm enough to pellet the cells before the agarose gelled. After 1 hr on ice, the gelled pellets were removed from the tubes and cut into 1-mm^3^ blocks in dH_2_O at room temperature. Blocks were placed in buffer at room temperature and then rinsed once with room temperature dH_2_O before en bloc staining with 1% uranyl acetate in dH_2_O in the dark for 1 hr at room temperature. Samples were rinsed once with room temperature dH_2_O and then dehydrated through a graded ethanol series (30%, 50%, 70%, 95%, 3× 100%) for 1 hr each change and then infiltrated with Spurr’s resin (1:1 Spurr’s:ethanol, 3:1 Spurr’s:ethanol, and three changes of 100% Spurr’s resin in vacuum) for at least 6 hr each. Individual pieces were embedded in fresh 100% Spurr’s in BEEM capsules at 70° for 2 d. Thin sections were cut with an LKB NOVA Ultrotome III (Leica, Brannockburn IL), collected on 200-mesh grids, and then stained with 4% aqueous uranyl acetate for 1 hr and Reynold’s lead citrate for 4 min. Grids were viewed using a JEOL 1200EX transmission electron microscope (JEOL USA Inc,, Peabody, MA). Images were recorded on Kodak 4489 film (Eastman Kodak Co., Rochester NY) that was then scanned using an Epson 4870 (Epson America, Inc. Long Beach, CA) flatbed scanner at 1200 dpi. Digital negatives were processed to positives and labeled using Photoshop CS4.

### Melanin production assay

Niger seed and L-DOPA plates were used to determine *C. gattii* melanin production. Strains were grown overnight at 24°, washed twice with dH_2_O, diluted to 1 OD_600_/mL, and then 5 µL of cell suspension was spotted on Niger seed and L-DOPA agar plates and incubated for 72 hr at 24°.

### Capsule production assay

Strains were cultured in YPD media overnight at 24°, washed twice with dH_2_O, and diluted to 0.2 OD_600_/ml (5 mL) in low iron media for growth at 24° for 72 hr. Three microliters of India ink (Gibson Laboratories, Inc) was added to 97 µL of cell suspension. The images were taken at 1000× magnification.

### Mouse infection studies

Animals studies conducted in the Division of Laboratory Animal Resources facilities at Duke University Medical Center (DUMC) were conducted in good practice as defined by the United States Animal Welfare Act and in full compliance with the guidelines of the DUMC Institutional Animal Care and Use Committee. The vertebrate experiments were reviewed and approved by the DUMC Institutional Animal Care and Use Committee under protocol number A217-11-08.

Seven- to eight-week-old female A/Jcr mice (Jackson Laboratory, 18−22 g) were used in this study. For infection, strains were cultured in YPD broth overnight at 24° and washed twice with sterile phosphate-buffered saline (PBS). Cells were counted with a hemocytometer and resuspended in sterile PBS at 10^6^ cells per milliliter. Dilutions of the cells were plated onto YPD and incubated at 24° for 48 hr to determine CFU and viability. Groups of 10 mice (excluding R265, which had eight mice; two mice died due to pentobarbital treatment) per strain were anesthetized with pentobarbital (Lundbeck Inc, Deerfield, IL), and inoculated with *C. gattii* via intranasal instillation of an inocula of 5 × 10^4^ cells (in 50 µL). Survival was monitored 1 to 2 times daily, and moribund mice were killed with CO_2_. Kaplan-Meier survival curves were generated with Prism 5.03 (GraphPad software, La Jolla, CA), and *P* values were evaluated by a Log-rank (Mantel-Cox) test. A *P* value of <0.05 was considered significant.

To determine fungal burden, the lungs and brains of *C. gattii* infected mice (n = 5 for each strain) were dissected at day 14 after infection. Half organ portions were weighed, transferred to a 15-mL Falcon tube filled with 5 ml PBS, and homogenized for 10 sec at 13,600 rpm/min (Power Gen 500, Fisher Scientific). Tissue homogenates were serially diluted, and 100 µL was plated onto a YPD plate containing 100 µg/mL chloramphenicol. The plates were incubated at 24° for 48 to 72 hr to determine CFU per gram of lung or brain. The significance of differences in fungal burden was determined using one-way analysis of variance (ANOVA) and Dunnett’s multiple comparison test. For histopathological analysis, half organ samples of lung and brain were fixed in 10% phosphate-buffered formalin (Fisher Scientific), and mucicarmine and hematoxylin & eosin (H&E) stainings were performed by technicians at the Department of Pathology at Duke University. After slide preparation, each sample was examined thoroughly by microscopy for analysis of *Cryptococcus* colonization (mucicarmine) and tissue necrosis (H&E). Images were captured using an Olympus Vanox microscope (PhotoPath; Duke University Medical Center).

### Wax moth infection studies

Wax moths (*Galleria mellonella*) of the final instar larval stage (~0.3 g) from Vanderhorst Wholesale, Inc. (St. Marys, OH) were used (10 per strain) within 7 d from the day of shipment. The larval infection protocol was adapted from previously described methods for *C. neoformans* (Mylonakis *et al.* 2005) with minor modifications. Each larva was infected with 5 × 10^4^ or 1 × 10^5^ *C. gattii* cells in 5 µL of PBS by injection into the last pseudopod followed by incubation at 24° in a Petri dish with wood shavings. Larvae showing signs of severe morbidity, such as changes in body color and no response to touch were killed by cold treatment at −20°. The number of surviving wax moths was monitored and recorded daily.

## Results

### Calcineurin is critical for thermotolerance of *C. gattii*

**Figure 1:**
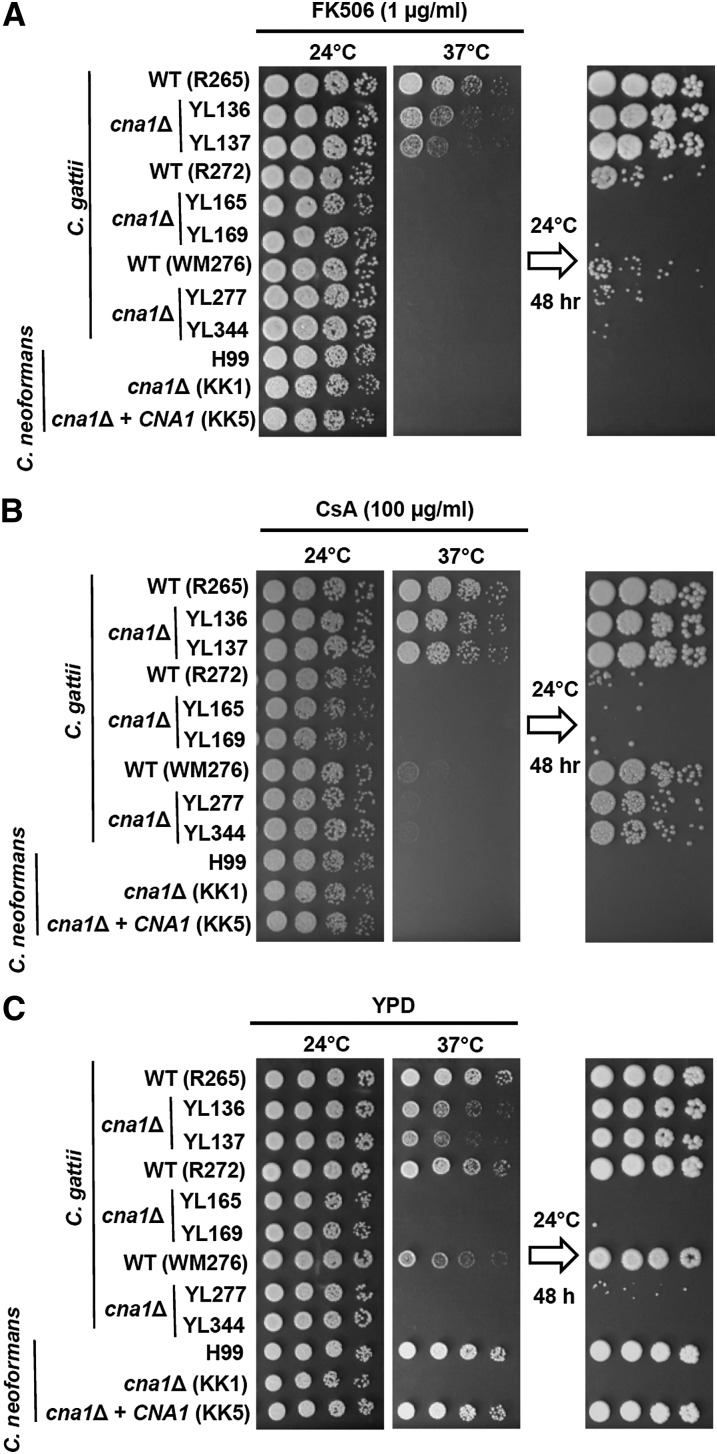
Calcineurin is critical for thermotolerance in *C. gattii*. (A and B) Inhibition of calcineurin via calcineurin inhibitors (FK506, CsA). Cells were grown overnight in YPD at 24°, fivefold serially diluted, spotted onto YPD medium containing FK506 (A) or cyclosporin A (CsA) (B), and incubated at the indicated temperatures for 48 hr. The plate incubated at 37° was transferred to 24° for 48-hr incubation to test viability of the strains after FK506 or CsA inhibition at 37°. (C) Deletion of *C. gattii* calcineurin catalytic A subunit results in temperature sensitivity. Cells were grown overnight in YPD at 24°, fivefold serially diluted, spotted onto YPD medium, and incubated at the indicated temperatures for 48 hr. The plate incubated at 37° was transferred to 24° for 48-hr incubation to test whether the temperature-sensitive calcineurin mutants survived exposure to the nonpermissive temperature.

The ability to grow at host body temperature (37°) is a critical virulence factor for pathogens to establish infection. Previous studies on Sod1, Tps1, Tps2, Pka1, Pka2, Ste12α, and Gat1 have revealed examples of divergent functions between *C. gattii* and *C. neoformans*. Here, we examined whether *C. gattii* calcineurin has divergent or conserved functions compared with *C. neoformans*. We first tested the effect of the calcineurin inhibitors FK506 and cyclosporin A (CsA) on *C. gattii* growth at room (24°) and body (37°) temperatures. We found that *C. gattii* R265, R272, WM276, and *C. neoformans* H99 wild-type strains exhibited different responses at 37° in the presence of FK506 or CsA (Figure 1, A and B). *C. gattii* R265 was more tolerant to growth at 37° in the presence of a calcineurin inhibitor compared with the R272, WM276, and H99 strains (Figure 1, A and B). This finding suggested that (1) calcineurin could be less important for growth at 37° in the *C. gattii* R265 isolate; (2) FK506 is either not efficiently imported into the cells, or is being rapidly exported; or (3) FKBP12 is not sufficiently abundant to inhibit all calcineurin activity.

To test these models and determine whether the phenotypes observed are due to pharmacological inhibition of calcineurin, we genetically disrupted the calcineurin catalytic subunit A gene from the three *C. gattii* isolates representing the VGIIa (R265), VGIIb (R272), and VGI (WM276) molecular genotypes. Although R265 *cna1*Δ mutants were more tolerant to growth on solid medium at 37° compared with *cna1*Δ mutants generated from *C. gattii* R272, WM276, and *C. neoformans* H99 strains (Figure 1C), the R265 *cna1*Δ mutants did exhibit a clear growth defect at 37° that was more severe than the modest impact of the calcineurin inhibitor FK506 or CsA on strain R265 growth at 37°. Thus, either drug import is limiting, drug export is more prominent, or FKBP12 or cyclophilin A is limiting. In addition, we noted that the R265 *cna1*Δ mutants exhibited growth defects in the presence of FK506 (but not CsA) compared with R265 wild-type in the presence of FK506 (Figures 1, A and B), providing evidence that FK506 may have target(s) in addition to calcineurin upon exposure to 37° in *C. gattii* R265.

To test whether loss of calcineurin function exerts a fungicidal effect at 37°, we shifted cells to 24° after exposure at 37°. We found that most calcineurin mutant cells from *C. gattii* R272, WM276, and *C. neoformans* H99 grown at 37° for 48 hr were inviable after transferring to 24° for 48 hr growth, whereas most calcineurin mutant cells from R265 were viable (Figure 1). Previous studies demonstrated that *Candida glabrata* calcineurin mutants are also temperature sensitive and the growth defects at elevated temperature can be rescued with an osmotic stabilizer (1 M sorbitol) (Chen *et al.* 2012). We then tested whether sorbitol can rescue growth defects of *C. gattii* and *C. neoformans* calcineurin mutants at 37°. We found that sorbitol could not rescue the growth defects of *C. gattii* or *C. neoformans* calcineurin mutants at 37° (data not shown), suggesting divergent roles of calcineurin for controlling osmotic responses between *Cryptococcus* species and *C. glabrata*.

**Figure 2:**
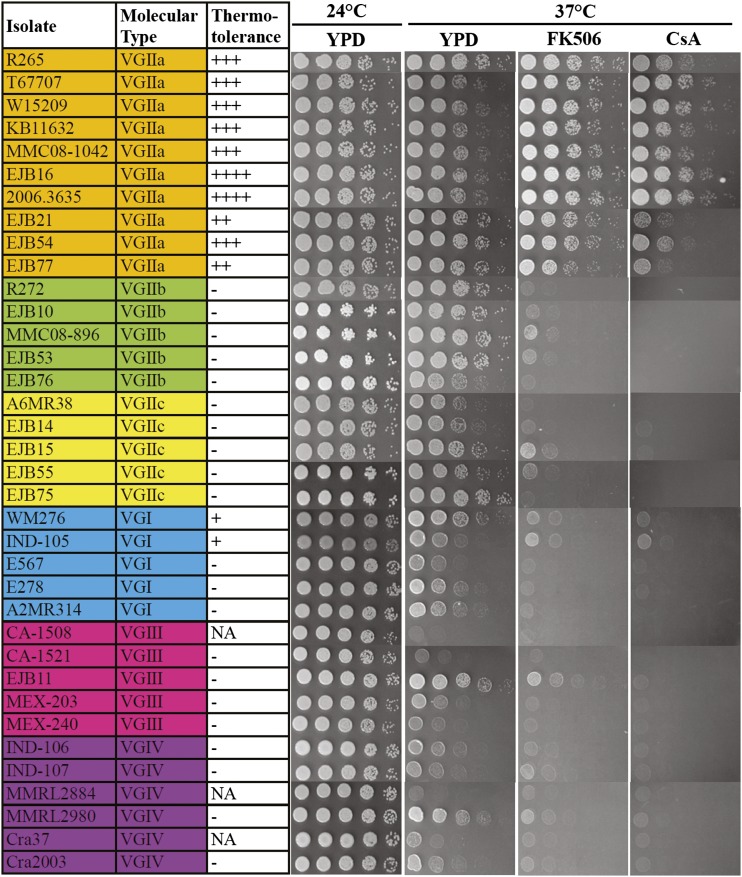
Molecular-type VGIIa isolates are more tolerant to calcineurin inhibitors at 37°. Thirty-six randomly selected *C. gattii* isolates covering four molecular types [VGI, VGII(a,b,c), VGIII, and VGIV] were grown overnight in YPD at 30°, fivefold serially diluted, spotted onto YPD medium in the absence or presence of FK506 (1 µg/mL) and CsA (100 µg/mL), and incubated for 48 hr at 24° and 37°. FK506 and CsA have no obvious effects on cryptococcal growth at 24°, and thus data are not shown. The calcineurin inhibitor tolerance of R265 was set as a control scale with three pluses (+++). The isolates that were hypersensitive to calcineurin inhibitor compared with R265 were labeled with ++ or + symbol, whereas isolates that were extremely sensitive to FK506 or CsA were given a – symbol, indicating that calcineurin is essential in the isolate. NA indicates not available due to the wild-type strain being hypersensitive to growth at 37° in the absence of either calcineurin inhibitor.

Roles of calcineurin in thermotolerance are distinct in the *C. gattii* outbreak isolate R265 compared with the *C. gattii* R272 and WM276 or *C. neoformans* H99 isolates (Figure 1). We hypothesize that tolerance for growth at 37° despite loss of calcineurin is specific to VGIIa. We therefore investigated 36 *C. gattii* isolates representing all four molecular types, VGI through VGIV. In VGII, representatives of the Pacific Northwest outbreak VGIIa/major, VGIIb/minor, and VGIIc/novel genotypes were also included. Our hypothesis is supported by the findings shown in Figure 2 as all VGIIa isolates (100%; 10/10) were more tolerant to growth on solid agar medium at 37° in the presence of FK506 or CsA compared with isolates of the VGI, VGIIb, VGIIc, VGIII, and VGIV molecular types, suggesting a divergent role for calcineurin in controlling thermotolerance among different molecular types of *C. gattii*.

**Figure 3:**
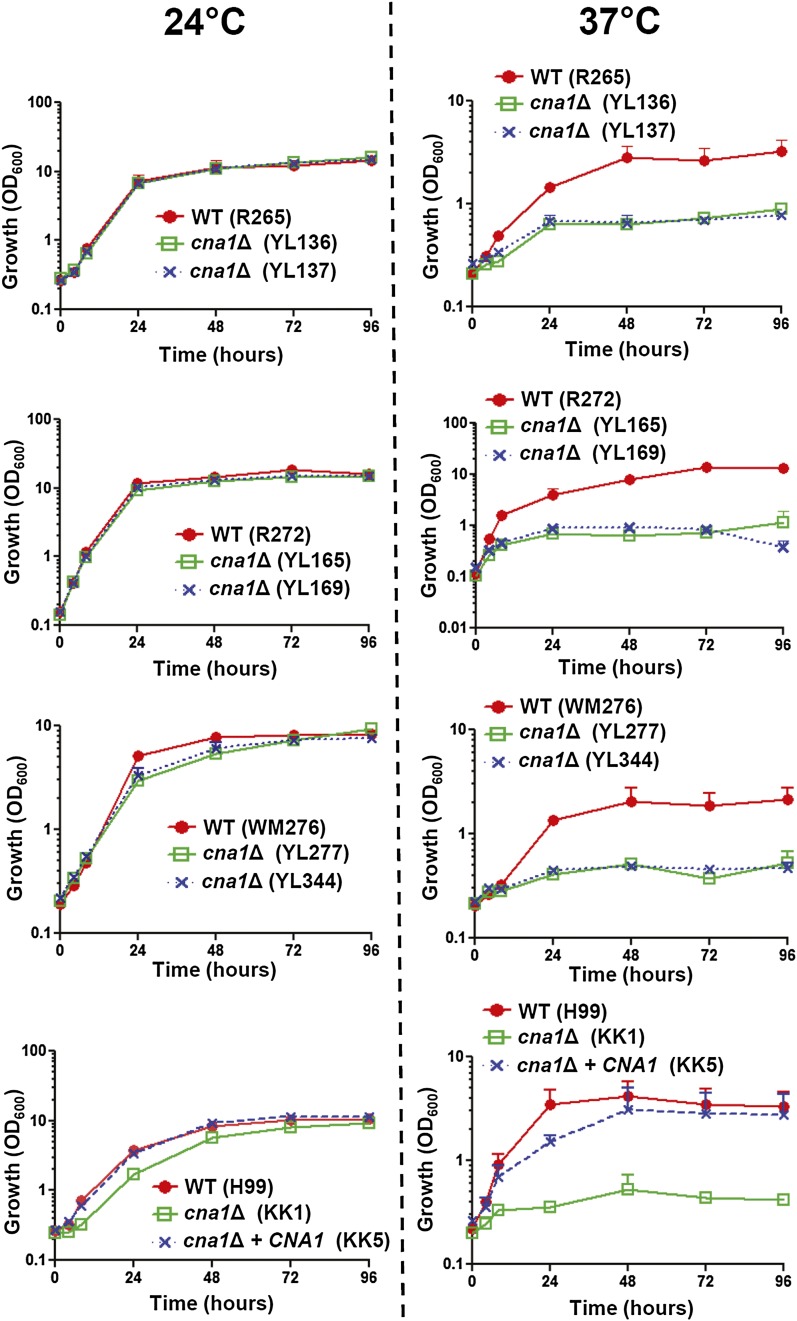
Growth kinetics of wild-type and calcineurin mutants in response to thermal stress in *C. gattii* and *C. neoformans*. Cells were grown overnight at 24°, washed twice with dH_2_O, diluted to 0.2 OD_600_/mL in fresh YPD medium, and incubated at 24° (left) and 37° (right) with shaking. The OD_600_ of cultures was measured at 0, 4, 8, 24, 48, 72, and 96 hr. The experiments were performed in triplicate, and data were plotted using Prism 5.03.

The growth kinetics (in liquid medium) of wild-type and calcineurin mutants from three *C. gattii* molecular type backgrounds were similar at 24°, whereas *C. neoformans* calcineurin mutants exhibited a slightly slower growth rate compared with the wild-type or complemented strains (Figure 3, left panel). At 37°, *C. gattii* and *C. neoformans* calcineurin mutants exhibited a slower growth rate compared with the wild-type (Figure 3, right panel), including calcineurin mutants of the VGIIa outbreak strain R265.

### Complementation of *cna1* mutant and isolation of suppressor mutations

Because R272 *cna1* mutants exhibit severe growth defects at 37°, this phenotype led us to isolate (1) *CNA1* complemented strains and (2) suppressed mutants via screening surviving colonies at a stringent *cna1* non-permissive condition (38°) after biolistic transformation. We used the R272 *CNA1* ORF flanked by ~1 kb of the 5′ NCR and 3′ NCR regions (~4.6 kb in total) to complement the *cna1* mutation in strain YL165 and isolated three types of transformants able to grow at 38°, including: *1) *CNA1* native-locus integration (YC875; *CNA1* positive, *NAT* negative), (2) *CNA1* ectopic integration (YC879; *CNA1* and *NAT* positive), and (3) suppressor mutations (YC883 and YC886; *CNA1* negative, *NAT* positive) (Figure S7).

Although all three were restored to growth at 37°, the three types of transformants exhibited different responses to SDS and fluconazole. In the native-locus complemented strain YC875 the growth defects conferred by the *cna1* mutation were fully rescued on YPD medium containing SDS or fluconazole, while ectopic complementation (strain YC879) partially rescued the growth defects of the *cna1* mutant on YPD medium containing SDS, but not fluconaole (Figure S7B). On the other hand, suppressor mutants YC883 and YC886 were only rescued for the growth defects conferred by the *cna1* mutation at 38° but not on YPD medium containing SDS or fluconazole. This suppressor analysis suggests a possible bifurcation of the calcineurin pathway, with one branch responding to temperature stress and another to cell wall/membrane stress. To decipher the nature of these suppressor mutations, we plan to conduct whole genome sequencing in the future.

### Calcineurin is essential for virulence in *C. gattii*

**Figure 4:**
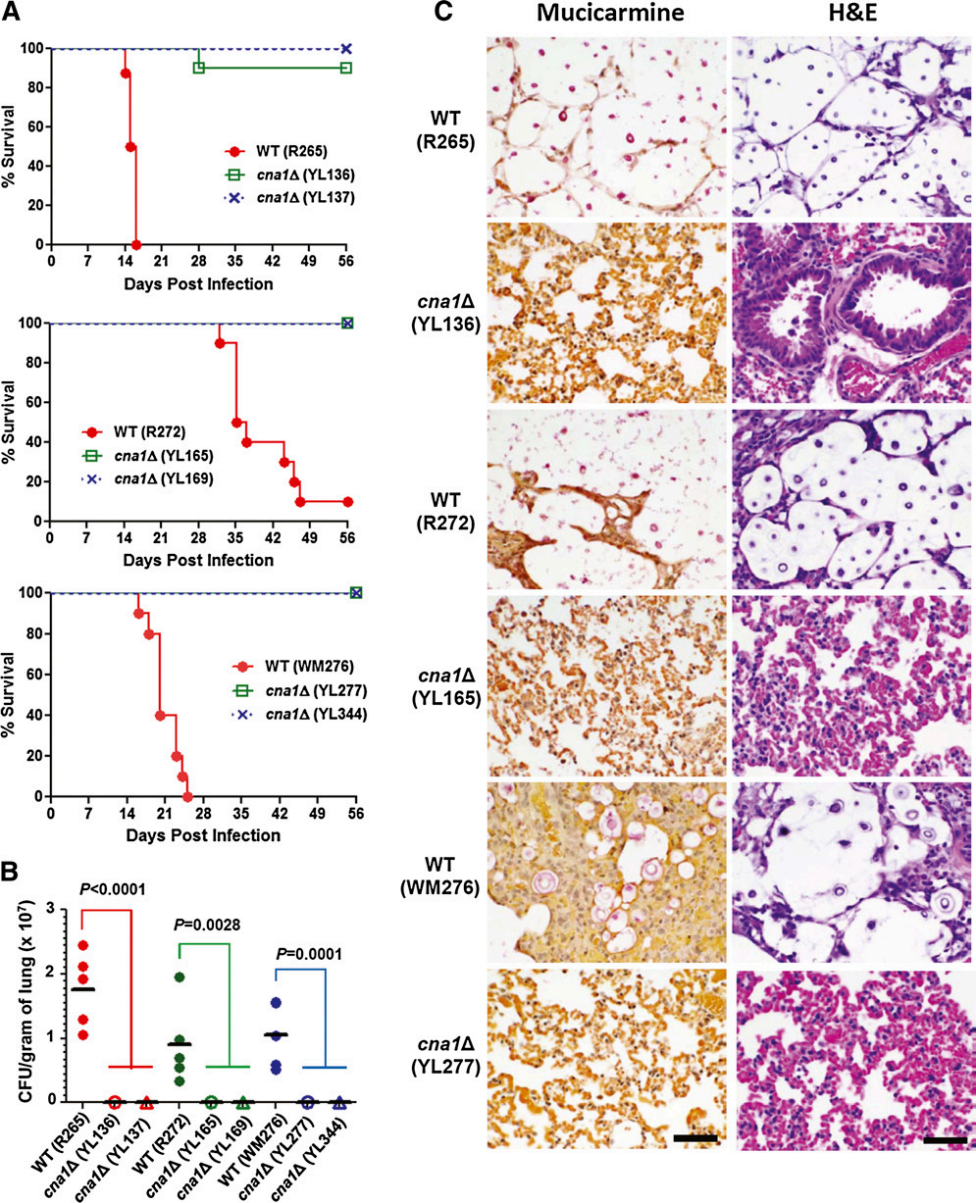
*C. gattii* calcineurin mutants are compromised for virulence in a murine inhalation infection model. (A) Groups of 10 A/Jcr mice were each infected via intranasal instillation with an infectious inocula of 5 × 10^4^ cells (in 50 µL) of the wild-type and two independent *cna1*Δ mutants. Survival percentage was monitored for 8 wk postinfection. (B) The fungal burden in the lungs was determined at 14 d postinfection (5 × 10^4^ cells per mouse). Five mice per strain were used. *P* values were determined by ANOVA and Dunnett’s multiple comparison test. (C) Histopathological sections of lungs dissected from mice infected with wild-type and mutant from R265, R272, and WM276 strains. The mice were infected with 5 × 10^4^ yeast cells and sacrificed at day 14. Mucicarmine and H&E stains were used to observe *C. gattii* colonization and tissue necrosis, respectively. Scale bar = 50 µm.

Given the growth defects of calcineurin mutants at mammalian body temperature, we tested the hypothesis that calcineurin would be required for *C. gattii* virulence in a murine inhalation model. Previous studies have shown that *C. neoformans* calcineurin is required for virulence in both rabbit and murine models (Cruz *et al.* 2000; Fox *et al.* 2001; Odom *et al.* 1997a). However, the roles of calcineurin in virulence have not yet been studied in *C. gattii*. We found that the three wild-type strains differ in pathogenicity. R265 VGIIa wild type (median survival: 15.5 days) is more virulent than both WM276 (median survival: 20 days; *P* = 0.0001; log-rank test) and R272 (median survival: 36 days; *P* < 0.0001), whereas WM276 is more virulent than the R272 strain (*P* < 0.0001; Figure 4A and Figure S4A). This finding suggests that a *C. gattii* environmental strain (WM276) can be as virulent as clinical isolates (R265 and R272). Because *C. gattii* calcineurin mutants from the three wild-type strains exhibit different responses to temperature, it was of interest to determine whether these mutants are still able to cause infection in a murine inhalation model. We found that *C. gattii* calcineurin mutants from all three molecular genotype isolates were avirulent or strongly attenuated for virulence (*P* < 0.0001 as compared to each wild type; Figure 4A).

The strongly attenuated virulence of calcineurin mutants might be due to reduced or loss of *in vivo* proliferation in the murine host. Studies in experimental pathobiology and clinical epidemiology have suggested that *C. gattii* preferentially causes pulmonary infection, whereas *C. neoformans* preferentially causes disease of the central nervous system (Ngamskulrungroj *et al.* 2012b; Perfect 2012; Sorrell *et al.* 2012). To determine colonization ability, we performed fungal burden analysis in the lungs and brains of animals infected with wild type and *cna1*Δ mutants. We found that calcineurin mutants exhibited strongly reduced fungal burden in the lung tissues compared with the respective wild type isolates (*P* < 0.01; ANOVA and Dunnett’s multiple comparison test; Figure 4B). Calcineurin mutants exhibited little or no fungal burden in lung tissues, whereas wild-type strains proliferated well (Figure 4B). With regard to brain tissue, we did not recover any calcineurin mutant cells from R265, R272, and WM276, whereas we were able to recover wild-type cells from several, but not all, infected mice (Figure S2).

Histopathological mucicarmine staining of tissues revealed that the wild-type R265, R272, and WM276 strains proliferated extensively in lung tissues, whereas calcineurin mutants from each wild type were not observed (Figure 4C; left panels), suggesting that calcineurin is required for proliferation in the lung. In H&E staining, alveolar damage or tissue necrosis in lung tissues was only observed in animals infected with the wild-type strains but not with the calcineurin mutants (Figure 4C; right panels). The histopathological analysis was well correlated with fungal burden in the lung tissues. We did not observe mucicarmine-stained or damaged tissues in the brain tissues of animals infected with any strain at the time point examined (data not shown). We further test whether *C. gattii* calcineurin is required for melanin and capsule production. We found that *C. gattii* calcineurin played no roles in melanin production (Figure S3 and File S1), but minor roles in capsule production (Figure S6).

### Calcineurin mutants exhibit multiple vesicle phenotypes and plasmamembrane disruptions upon thermal stress in *C. gattii* and *C. neoformans*

**Figure 5:**
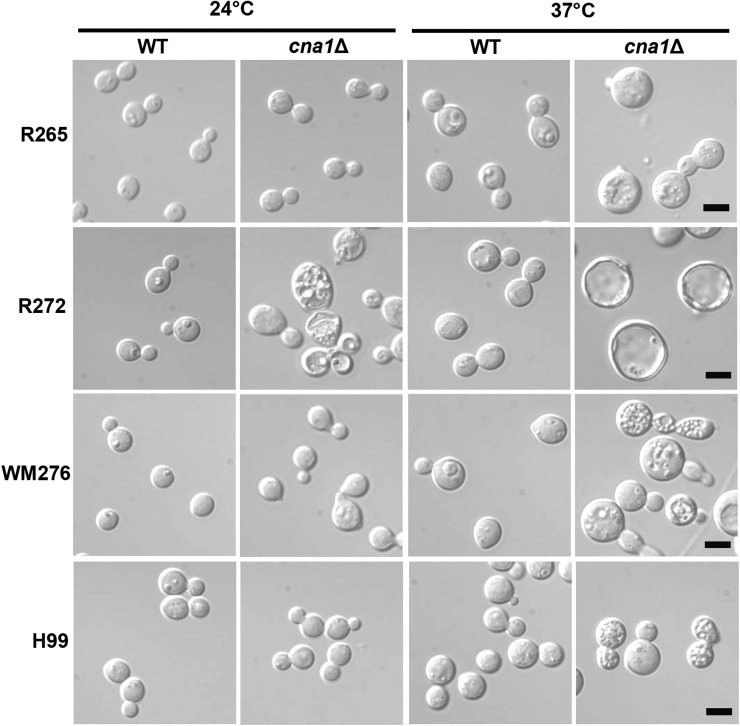
Calcineurin mutants exhibit multivesicular phenotypes at 37°. Strains were grown in liquid YPD medium overnight at 24°. Cells were washed twice, diluted into two tubes of 0.4 OD_600_/mL, and incubated for 5 hr at 24° and 37° to reach log phase. The cells were spun and washed twice with PBS buffer, then resuspended in 5 mL of fixative buffer [1:1 ratio of 0.2 M Sodium cacodylate (pH = 6.8) and 6% glutaraldehyde in dH_2_O]. The *cna1*Δ mutants tested were YL136 (from R265), YL165 (from R272), YL277 (from WM276), and KK1 (from H99). Cells were viewed by light microscopy at 1000× magnification and photographed. Scale bar = 5 µm.

We investigated whether calcineurin mutants exhibit morphological changes when exposed to 24° and 37°. We found that *C. gattii* calcineurin mutants from R265 and WM276 and *C. neoformans* H99 exhibited multiple vesicle phenotypes at 37°, whereas these mutants exhibited a normal wild-type morphology at 24° (Figure 5). Interestingly, *C. gattii* calcineurin mutants from the R272 isolate exhibited more severe phenotypes such as larger ring-like cells in addition to multiple vesicle phenotypes at 37° (Figure 5). In contrast to calcineurin mutants from *C. gattii* R265 and WM276, and *C. neoformans* H99 isolates, *C. gattii* calcineurin mutants from R272 also showed multiple vesicle phenotypes at 24° (Figure 5).

**Figure 6:**
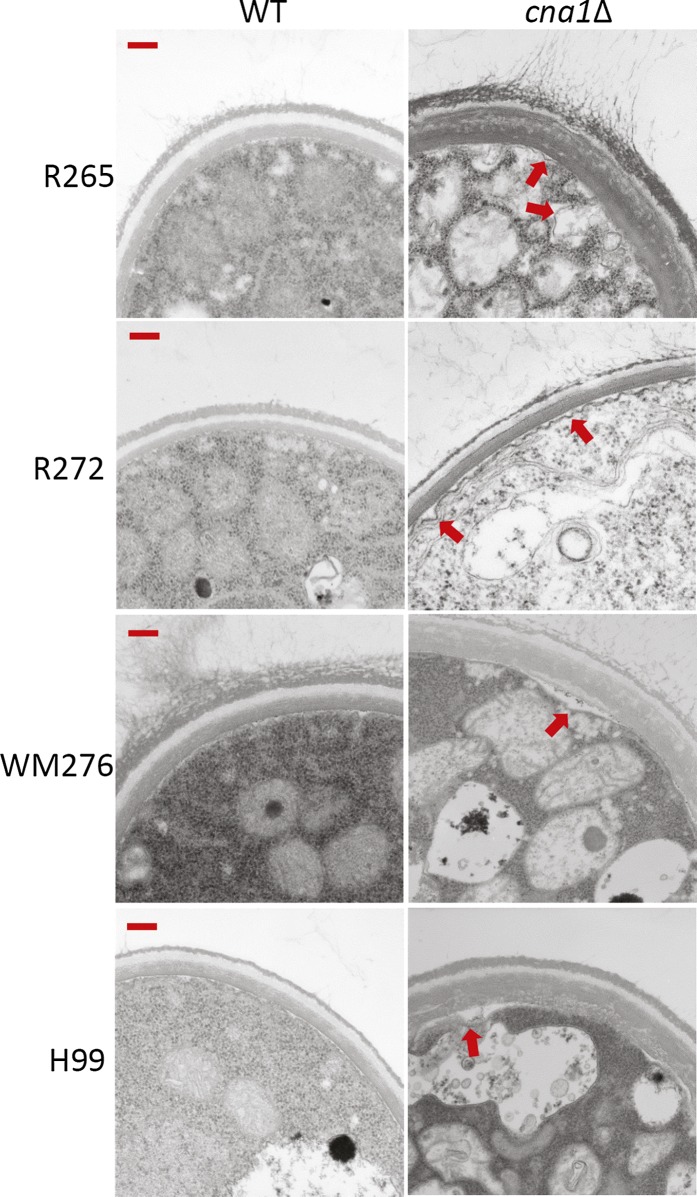
Calcineurin mutants exhibit plasma membrane disruptions at 37° (indicated by red arrows). Cell growth and fixation conditions are described in *Materials and Methods*. Transmission electron microscopy was used to observe intracellular architecture. Only cells grown at 37° are shown. Images were taken at 25,000× magnification. Scale bar = 200 nm.

The intracellular defects of *C. neoformans* calcineurin mutants have not been investigated (Kojima *et al.* 2006; Odom *et al.* 1997b). We therefore examined intracellular defects via transmission electron microscopy of *C. gattii* and *C. neoformans* calcineurin mutants exposed to 24° and 37°. We found that *C. gattii* and *C. neoformans* calcineurin mutants exhibited plasma membrane disruptions at 37° (Figure 6, right panels) but not at 24° (data not shown), whereas wild-type isolates had intact plasma membranes (Figure 6, left panels).

### Divergent roles of calcineurin in cell membrane and cell wall integrity and endoplasmic reticulum (ER) stress responses

**Figure 7:**
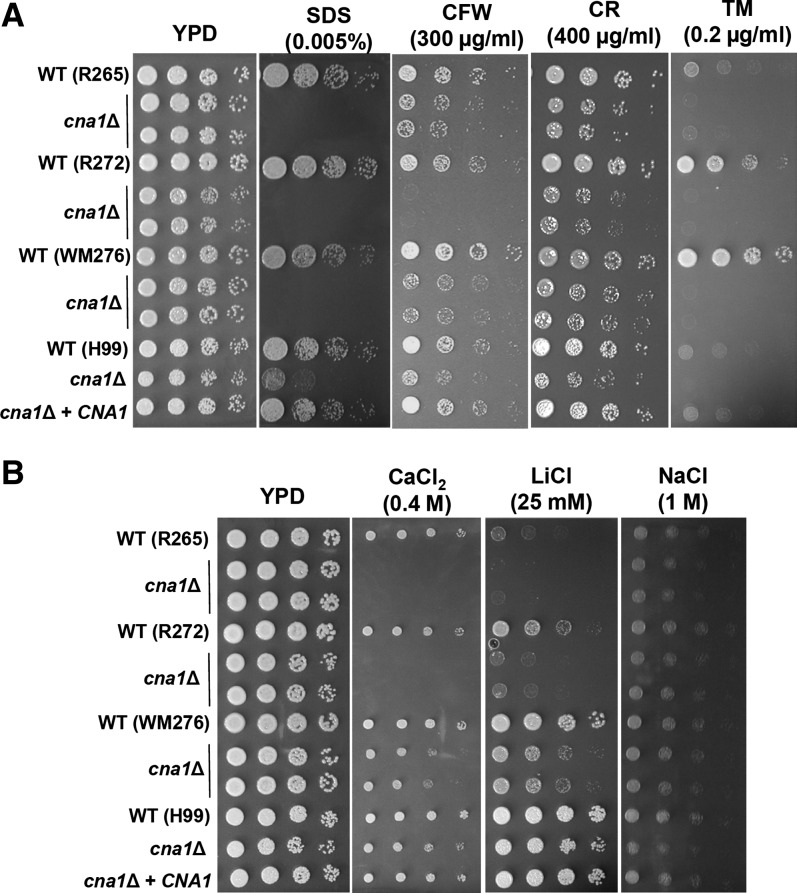
Divergent roles of calcineurin in cell wall integrity, ER stress response, and cation homeostasis. (A) Cells were grown overnight in YPD at 24°, fivefold serially diluted, and spotted onto YPD medium containing SDS, calcofluor white (CFW), Congo red (CR), and tunicamycin (TM) at the indicated concentrations and incubated at 24° for 48 hr. (B) In *C. gattii* calcineurin plays a larger role than in *C. neoformans* in controlling cation homeostasis. Cells were grown overnight in YPD at 24°; fivefold serially diluted; spotted onto YPD medium containing CaCl_2_, LiCl, or NaCl at the indicated concentrations; and incubated at 24°C for 48 hr.

The roles of *C. neoformans* calcineurin on cell membrane and cell wall integrity remain elusive (Kojima *et al.* 2006; Odom *et al.* 1997b). Here, we demonstrate that in *C. gattii* and *C. neoformans*, calcineurin is required for cell membrane integrity as evidenced by the fact that calcineurin mutants are more sensitive to sodium dodecyl sulfate (SDS) (Figure 7A), a reagent which compromises cell membrane integrity. In *C. gattii* and *C. neoformans* calcineurin plays minor roles in response to the cell wall-disturbing agents calcofluor white and Congo red compared with SDS (Figure 7A).

Recently, Kozubowski *et al.* demonstrated that *C. neoformans* calcineurin (Cna1) interacts with the COPI component Sec28 and the COPII component Sec13 (Kozubowski *et al.* 2011), which are involved in Golgi-to-ER protein traffic and vesicle formation during ER-to-Golgi transport, respectively. We were interested in investigating the roles of calcineurin in response to the ER stress inducer tunicamycin, a chemical that blocks the synthesis of N-linked glycoproteins and induces the unfolded protein response. We found that in *C. gattii* and *C. neoformans* calcineurin is essential for ER stress responses because calcineurin mutants are sensitive compared to wild-type isolates (Figure 7A). Interestingly, we found that *Cryptococcus* wild-type isolates tolerate tunicamycin differently, with *C. gattii* WM276 > R272 > R265 = *C. neoformans* H99 (Figure 7A), indicating that the unfolded protein response pathway might be evolutionary diverged between *C. gattii* and *C. neoformans*.

### Divergent roles of calcineurin in Ca^2+^ and Li^+^ homeostasis

The role of *C. neoformans* calcineurin in response to Ca^2+^ cation remains elusive (Cruz *et al.* 2000; Kojima *et al.* 2006; Odom *et al.* 1997b). Calcineurin plays a role as a positive regulator of Ca^2+^ tolerance in *C. albicans*, *C. dubliniensis*, and *C. lusitaniae* (Chen *et al.* 2011; Sanglard *et al.* 2003; Zhang *et al.* 2012b), whereas it acts as a negative regulator of Ca^2+^ tolerance in *S. cerevisiae* and *C. glabrata* (Chen *et al.* 2012; Withee *et al.* 1998), suggesting divergent functions of calcineurin in controlling Ca^2+^ homeostasis in fungi. We demonstrated that *C. gattii* requires calcineurin for optimal growth in the presence of Ca^2+^ (Figure 7B). In contrast to *C. gattii* calcineurin mutants, those of *C. neoformans* do not show obvious growth defects in response to Ca^2+^ compared with the wild type (Figure 7B), indicating a divergent role of calcineurin in controlling Ca^2+^ homeostasis between *C. gattii* and *C. neoformans*. Here, we also examined the role of calcineurin in response to Li^+^ cations and found that in both *C. gattii* and *C. neoformans* calcineurin plays a role similar to the Ca^2+^ response (Figure 7B), indicating that individual *C. gattii* or *C. neoformans* isolates have similar mechanisms in controlling Ca^2+^ and Li^+^ homeostasis. A previous study has been shown that calcineurin is required for Na^+^ homeostasis in *C. neoformans* (Cruz *et al.* 2000). Our data revealed that calcineurin plays a minor role in controlling Na^+^ homeostasis of *C. neoformans* H99 whereas calcineurin is not required for Na^+^ homeostasis in *C. gattii* R265, R272, and WM276 (Figure 7B).

### Divergent roles of *C. gattii* and *C. neoformans* calcineurin in fluconazole tolerance

**Figure 8:**
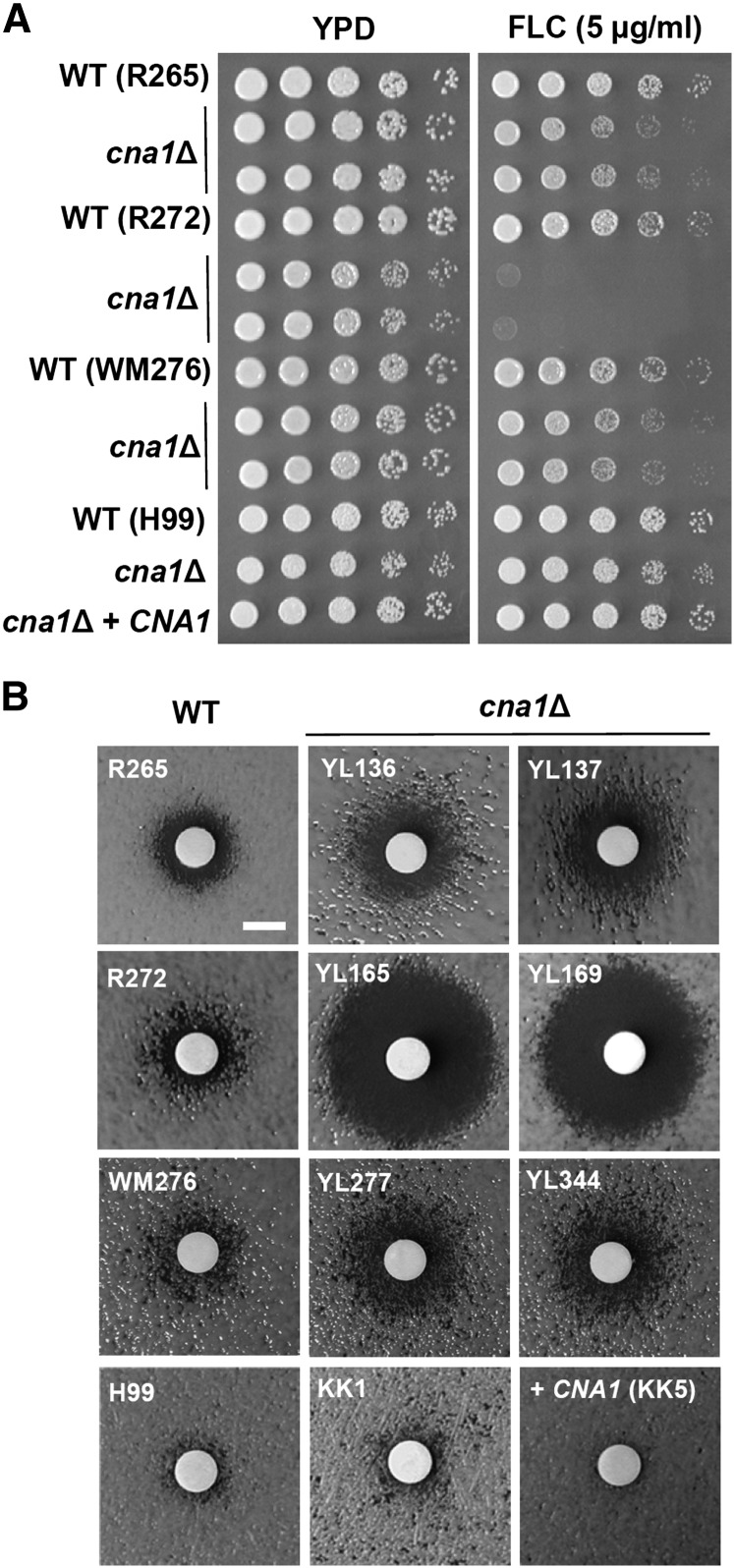
Divergent roles of calcineurin in fluconazole tolerance between *C. gattii* and *C. neoformans*. (A) Cells were grown overnight in YPD medium at 24°, fivefold serially diluted, and spotted onto YPD medium in the absence or presence of fluconazole (5 µg/mL). The plates were incubated at 24° for 48 hr. (B) Disk diffusion halo assays were used to determine fluconazole susceptibility of wild-type and mutant strains. Cells were grown overnight at 24°, and 100 µL of 0.1 OD_600_/mL was spread on the surface of YPD medium. A disk was placed on the surface of the medium, and fluconazole (20 µg) was added to each disk. The plates were incubated at 24° for 48 hr and photographed. Scale bar = 6 mm.

The primary therapy for patients with cryptococcal meningoencephalitis is amphotericin B (0.7 ~1 mg/kg/day, intravenously) plus flucytosine (100 mg/kg/day, orally) in four divided doses for at least 2 wk, followed by fluconazole (6 mg/kg/day, orally) for a minimum of 8 wk (Perfect *et al.* 2010). Although calcineurin is required for fluconazole tolerance in *Candida* species (Chen *et al.* 2011, 2012; Cruz *et al.* 2002), its role remains unclear in *C. neoformans*. Del Poeta *et al.* (2000) reported that FK506 exhibits *in vitro* synergistic antifungal activity with fluconazole against *C. neoformans* wild-type H99, the *cna1*Δ mutant, and the *frr1*Δ mutant (as evidence of FIC = 0.25), suggesting that FK506 shows synergistic antifungal activity with fluconazole via a mechanism that is independent of the FK506 target proteins calcineurin and FKBP12 (Del Poeta *et al.* 2000). Here, we demonstrate that *C. neoformans* H99 calcineurin is not required for fluconazole tolerance, as shown by the fact that calcineurin mutants exhibited similar growth via spotting assays (Figure 8A), inhibition halos via disk diffusion assays (Figure 8B), or MIC values via E-test (Table 2) compared with the wild-type in the presence of fluconazole. In contrast to *C. neoformans* calcineurin, we found that *C. gattii* calcineurin plays a greater role in fluconazole tolerance, especially in the R272 background, as evidenced by the fact that calcineurin mutants exhibited strong growth defects in spotting assays (Figure 8A), larger inhibition halos in disk diffusion assays (Figure 8B), and an eightfold increased fluconazole susceptibility compared with the wild type (Table 2). Calcineurin mutants from the *C. gattii* R265 and WM276 strains exhibited modest sensitivity to fluconazole compared with their corresponding wild-type isolates (Figure 8 and Table 2).

**Table 2:** Drug susceptibility of wild-type and calcineurin mutants of *C. gattii* and *C. neoformans*

Strains	Fluconazole	Voriconazole	Amphotericin B	Caspofungin
WT (R265)	32~48*^a^*	0.38	0.125~0.19	>32
* cna1*Δ (YL136)	16	0.125	0.38	>32
* cna1*Δ (YL137)	16	0.125	0.38	>32
WT (R272)	64	0.25	0.25	>32
* cna1*Δ (YL165)	8	0.064	0.38	>32
* cna1*Δ (YL169)	8	0.064	0.38	>32
WT (WM276)	32~48	0.25	0.25	>32
* cna1*Δ (YL277)	16	0.125	0.38	>32
* cna1*Δ (YL344)	16	0.125	0.38	>32
WT (H99)	128	0.5	0.25	>32
* cna1*Δ (KK1)	96	0.25	0.38	>32
* cna1*Δ + *CNA1* (KK5)	96	0.5	0.25	>32

WT, wild type; YPD, yeast extract peptone dextrose; dH_2_O, distilled water; OD, optical density.

a Cells were grown overnight in YPD and washed twice with sterile dH_2_O before diluting to 1 OD_600_/mL. A total of 0.5 mL (= 0.5 OD) of cells were spread on RPMI-1640 medium (Remel; w/MOPS and 2% glucose; pH 7.3 ± 0.1) and incubated at 24° for 48 hr before reading the E-test values. The numbers indicate minimum inhibitory concentrations (µg/mL).

## Discussion

### Roles of calcineurin in thermotolerance and virulence in *C. gattii* and *C. neoformans*

In this study, we demonstrate that *C. gattii* requires calcineurin (Cna1) for thermotolerance and virulence in a murine inhalation model. Although calcineurin mutants from *C. gattii* R265 are more tolerant to growth at 37° (Figure 1), 38°, and 39° (data not shown) on solid medium compared with mutants from R272 and WM276, they exhibited similar sensitivity at 37° in liquid medium compared with calcineurin mutants from R272 and WM276 (Figure 3). The growth defects of *C. gattii* calcineurin mutants at 37° were strongly correlated with attenuated virulence, suggesting that *C. gattii* uses calcineurin for survival and infection at body temperature. Therefore, the mechanism via which calcineurin promotes pathogenicity involves thermotolerance and is conserved between *C. gattii* and *C. neoformans*. Similarly, previous studies have shown that *Candida glabrata* and the protozoan parasite *Leishmania major* use calcineurin to cause infection via a thermotolerance mechanism (Chen *et al.* 2012; Naderer *et al.* 2011), suggesting convergent evolution of calcineurin roles in thermotolerance and virulence in basidiomycete and ascomycete yeast pathogens as well as a common parasite. However, in contrast to *Cryptococcus* species and *C. glabrata*, other pathogenic fungi use calcineurin in different mechanisms for pathogenicity: *C. albicans* (serum survival), *C. dubliniensis* (hyphal growth), *C. lusitaniae* (serum growth), *A. fumigatus* (filamentous growth), and *M. oryzae* (appresorial formation) (Chen *et al.* 2011; Choi *et al.* 2009; Cruz *et al.* 2002; Steinbach *et al.* 2006; Zhang *et al.* 2012b), suggesting divergent roles of fungal calcineurin for pathogenesis.

### Other mechanisms that *C. gattii* and *C. neoformans* calcineurin may employ during infection

Because calcineurin mutants have pleotropic phenotypes linked to virulence, *C. gattii* and *C. neoformans* might use calcineurin via other functions that enable pathogenicity. For example, *C. gattii* calcineurin mutants exhibit defects in cell membrane and cell wall integrity, as well as in ER stress responses (Figure 7). The fungal cells with defects in cell wall integrity and/or ER stress responses often exhibit strongly attenuated virulence in murine infection models (Chen *et al.* 2010b; Cheon *et al.* 2011). The wax moth infection model has been widely used as an alternative heterologous host virulence model for cryptococcal infection (Mylonakis *et al.* 2005). We demonstrated that *C. gattii* R265, R272, and WM276 wild-type strains exhibited similar virulence between murine inhalation and wax moth infection models (Figure S4). Using a wax moth infection model incubated at 24°, we demonstrated that *C. gattii* calcineurin is required for virulence in the R265 and WM276 strain background (Figure S5; *P* < 0.01; log-rank test), indicating that calcineurin also employs a mechanism other than thermotolerance for pathogenicity in the wax moth model. Interestingly, in the *C. gattii* R272 background calcineurin is only required for virulence in murine inhalation (37°), but not in a wax moth model (24°) (Figure S5), indicating that in *C. gattii* R272 the role of calcineurin in thermotolerance may be the major mechanism promoting infection in the murine inhalation model. However, the fact that the overall level of virulence of the R272 strain is reduced in the wax moth is a caveat to this conclusion.

### Role of calcineurin in azole tolerance of *C. gattii*

Azoles target fungal 14α-demethylase encoded by *ERG11*, which is involved in ergosterol biosynthesis and cell membrane/wall integrity (Vanden Bossche *et al.* 1998). Cell membrane perturbation by fluconazole can enhance uptake and toxicity of calcineurin inhibitors in *Candida albicans* (Cruz *et al.* 2002). Conversely, it is also possible that cell membrane defects caused by the *cna1*Δ mutation results in increased azole uptake, leading to synergistic fungicidal activity. Calcineurin has been demonstrated to be required for azole tolerance and/or resistance in the ascomycetous yeast pathogens *C. albicans*, *C. dubliniensis*, *C. glabrata*, and *C. lusitaniae* (Chen *et al.* 2011, 2012; Sanglard *et al.* 2003; Zhang *et al.* 2012a,b), but whether calcineurin functions in an analogous role in the basidiomycetous yeasts *C. neoformans* and *C. gattii* has been less clear. Previous studies demonstrated that the calcineurin inhibitor FK506 exhibits synergistic antifungal activity with fluconazole against *C. neoformans* strain H99, but the synergistic mechanism was found to be FKBP12- and calcineurin-independent (Del Poeta *et al.* 2000), indicating that FK506 might have additional targets in *C. neoformans*. Our data demonstrating that calcineurin is required for azole tolerance in *C. gattii* but not in *C. neoformans* support the findings by Del Poeta *et al.* (2000) and provide evidence for a divergent role of calcineurin in azole tolerance between *C. gattii* and *C. neoformans*. Even in *C. gattii*, three molecular types (VGIIa, VGIIb, and VGI) exhibit different levels of azole tolerance. For example, R272 *cna1*Δ mutants are hypersensitive to fluconazole compared with R265 and WM272 *cna1*Δ mutants (Figure 8 and Table 2). Because R272 *cna1*Δ mutants exhibit hypersensitivity to cell membrane/wall disturbing agents (Figure 7) that is correlated with increased azole susceptibility (Figure 8), we suggest that cell membrane defects caused by *cna1*Δ mutation may result in azole hypersensitivity.

In summary, our data suggest that in *C. gattii* calcineurin promotes thermotolerance and other mechanisms during infection in both the murine inhalation and wax moth models, in addition to a requirement for calcineurin in azole tolerance of *C. gattii*. Therefore, our studies provide support for *C. gattii* calcineurin as an attractive target for developing calcineurin inhibitors that retain antifungal activity but are reduced for immunosuppressive activity.

## Supplementary Material

Supporting Information
