# Supplementary material for: Calcineurin Governs Thermotolerance and Virulence of Cryptococcus gattii
Source: G3 (Bethesda). 2013 Mar 1;3(3):527–39. doi: 10.1534/g3.112.004242 (PMC3583459; doi:10.1534/g3.112.004242)
Supplement: Supporting Information [file supp_3_3_527__index.html]

Supporting Information 

# Calcineurin Governs Thermotolerance and Virulence of *Cryptococcus gattii*

## Supporting Information for Chen *et al.*, 2013

**Files in this Data Supplement:**

- Supporting Information - Figures S1-S7, File S1, and Table S1 (PDF, 864 KB)
- Figure S1 - Amino acid identity and pairwise alignments of calcineurin catalytic subunit (Cna1) from *C. gattii* R265, *C. gattii* WM276, and *C. neoformans* H99 (PDF, 278 KB)
- Figure S2 - The fungal burden of *C. gattii* wild-type and calcineurin mutants in the brain (PDF, 168 KB)
- Figure S3 - Calcineurin is not required for melanin production in *C. gattii* and *C. neoformans*. (PDF, 135 KB)
- Figure S4 - Comparison of *C. gattii* wild-type virulence in both murine inhalation and wax moth models (PDF, 182 KB)
- Figure S5 - Roles of *C. gattii* calcineurin in the wax moth model (PDF, 234 KB)
- Figure S6 - Calcineurin plays minor roles in capsule production. (PDF, 155 KB)
- Figure S7 - Complementation of R272 *cna1* mutant and isolation of suppressor mutations at 38°C (PDF, 160 KB)
- File S1 - Supplemental Results (PDF, 89 KB)
- Table S1 - PCR primers used in this study (PDF, 76 KB)
