# Supplementary material for: Calcineurin Governs Thermotolerance and Virulence of Cryptococcus gattii
Source: G3 (Bethesda). 2013 Mar 1;3(3):527–39. doi: 10.1534/g3.112.004242 (PMC3583459; doi:10.1534/g3.112.004242)
Supplement: Supporting Information [file supp_3.3.527_004242SI.pdf]

G3: Genes | Genomes | Genetics

Calcineurin governs thermotolerance and virulence of *Cryptococcus gattii*

Ying-Lien Chen[1,#], Virginia N. Lehman[1, 2], Yonathan Lewit[1], Anna F. Averette[1], and Joseph Heitman[1,*]

[1]Department of Molecular Genetics and Microbiology, Duke University Medical Center, Durham, North Carolina, USA
[2]Department of Biomedical Engineering, Duke University, Durham, North Carolina, USA

[*]Corresponding author: Joseph Heitman, MD, PhD
Mailing address: Department of Molecular Genetics and Microbiology,
Duke University Medical Center,
Durham, NC 27710, USA
Phone: (919) 684-2824, FAX: (919) 684-5458, Email: heitm001@duke.edu
[#] Present address: Department of Plant Pathology and Microbiology, National Taiwan University, 106 Taipei, Taiwan.

DOI: 10.1534/g3.112.004242

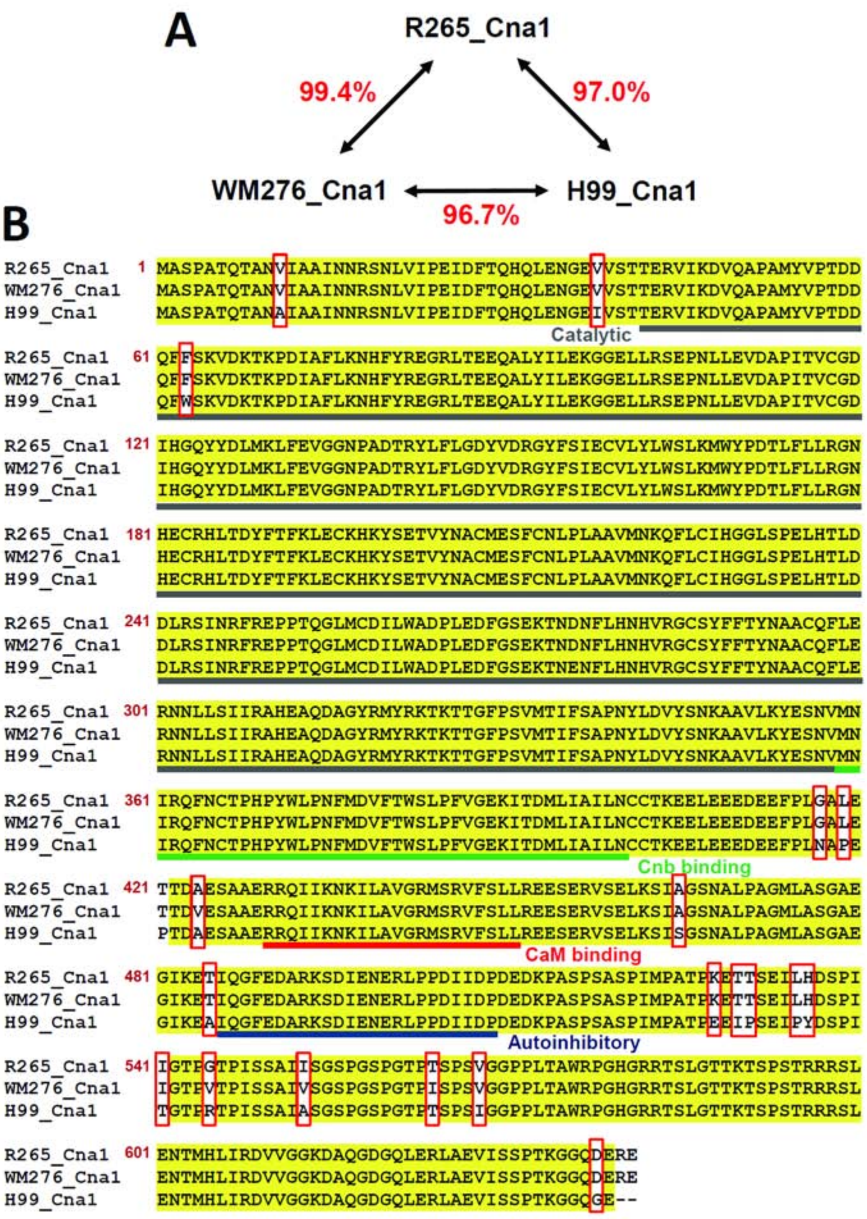

Figure S1 Amino acid identity and pairwise alignments of calcineurin catalytic subunit (Cna1) from *C. gattii* R265, *C. gattii* WM276, and *C. neoformans* H99. Amino acid identity and multiple sequence alignments are depicted using ClustalW software (http://npsa-pbil.ibcp.fr/cgi-bin/npsa_automat.pl?page=npsa_clustalw.html). **(A)** The % identity shared between the full-length proteins is shown in red. **(B)** The conserved and divergent amino acids are indicated with yellow shading and red rectangle, respectively. The catalytic domain, calcineurin regulatory subunit (Cnb) binding, calmodulin (CaM) binding, and autoinhibitory domains are indicated with gray, green, red, and blue underlining, respectively. The amino acids of the *C. gattii* R265 Cna1 protein are numbered.

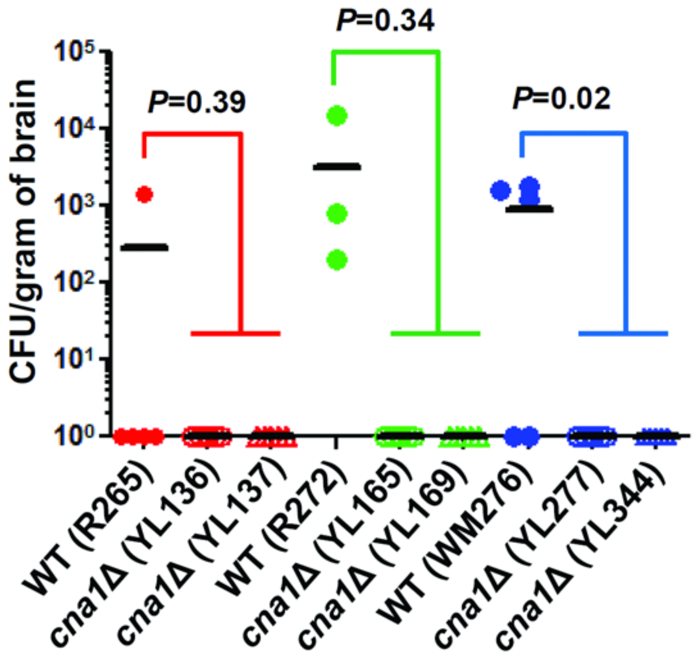

Figure S2 The fungal burden of *C. gattii* wild-type and calcineurin mutants in the brain. The fungal burden in the brain was determined at 14 days post-infection ($5x10^4$ cells per mouse). Five mice per strain were used. *P* values were determined by ANOVA and Dunnett's multiple comparison tests.

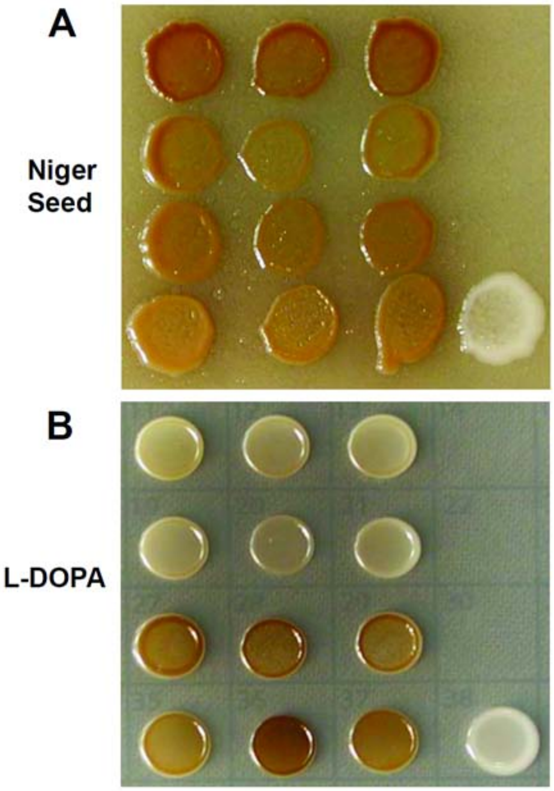

C

R265	*cna1*Δ (YL136)	*cna1*Δ (YL137)	
R272	*cna1*Δ (YL165)	*cna1*Δ (YL169)	
WM276	*cna1*Δ (YL277)	*cna1*Δ (YL344)	
H99	*cna1*Δ (KK1)	*cna1*Δ + *CNA1* (KK5)	*lac1*

Figure S3 Calcineurin is not required for melanin production in *C. gattii* and *C. neoformans*. Cells were grown overnight at 24°C, washed twice with dH_2O, diluted to 1 OD_{600}/ml, and 3 µl of cell suspension was plated on Niger seed **(A)** and L-DOPA **(B)** agar medium and incubated for 72 hr at 24°C. Strains analyzed are indicated in panel (**C**).

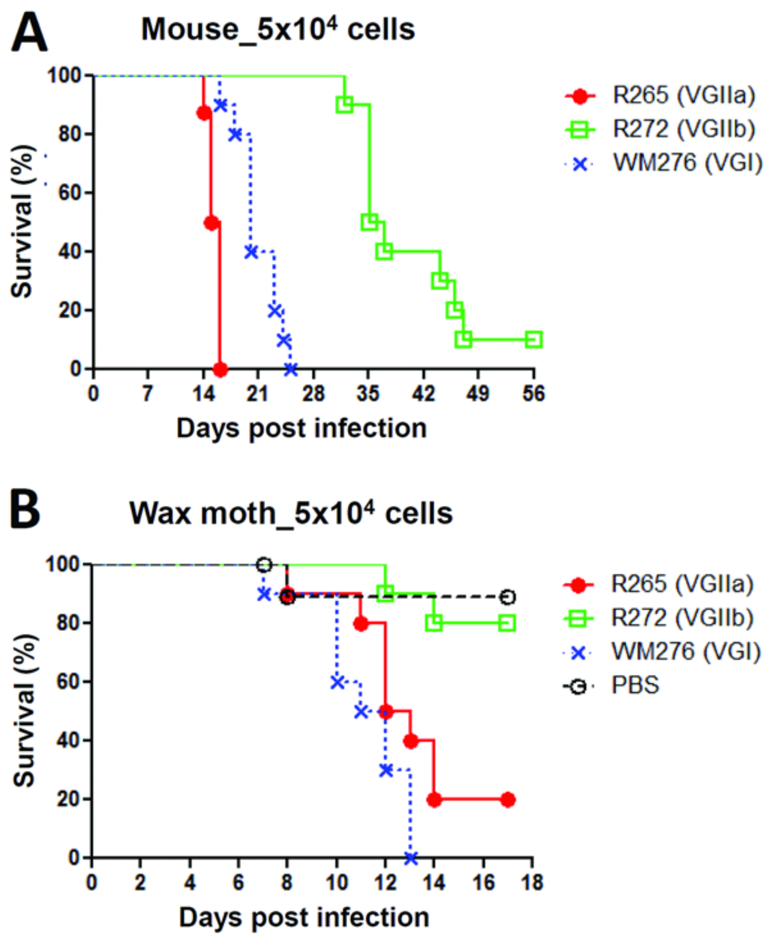

Figure S4 Comparison of *C. gattii* wild-type virulence in both murine inhalation and wax moth models. The data of *C. gattii* wild-type virulence in the murine inhalation (A) and wax moth models (B) were extracted from Figure 4 and Figure S5. Each mouse or wax moth received $5x10^4$ *C. gattii* yeast cells.

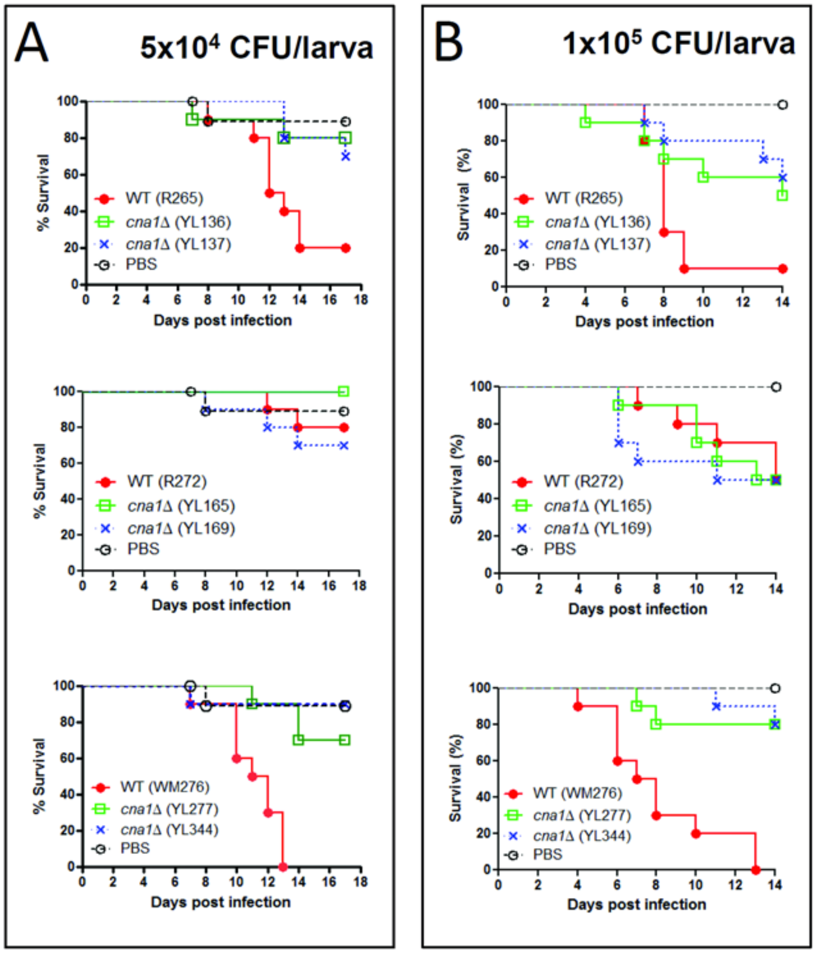

Figure S5 Roles of *C. gattii* calcineurin in the wax moth model. Survival of *Galleria mellonella* after injection of 5x10^{4} **(A)** or 10^{5} **(B)** CFU/larva of *C. gattii* wild-type or calcineurin mutants (10 moths per isolate).

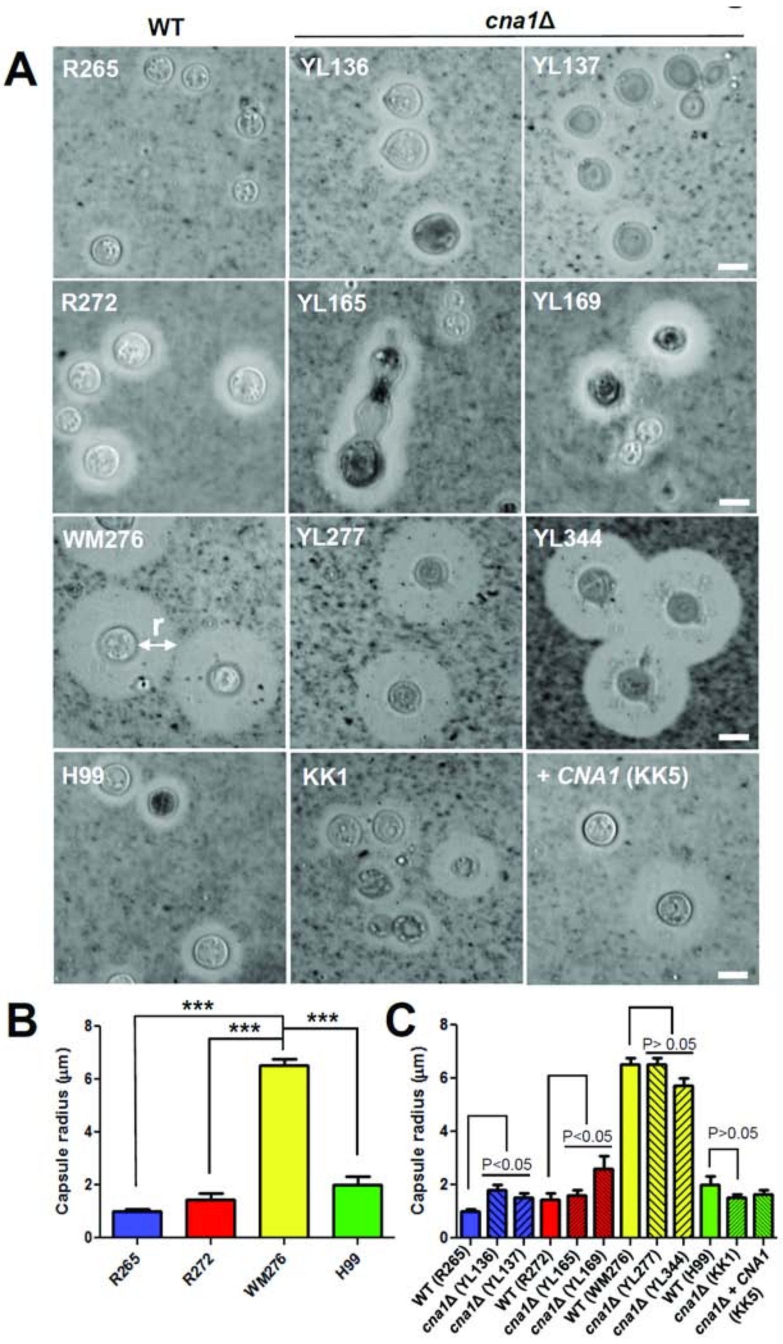

Figure S6 Calcineurin plays minor roles in capsule production.

(A) Capsule radius (labeled with r) of wild-type and calcineurin mutants was determined with India ink staining. Cells were grown overnight at 24°C in YPD media. Cells were washed twice with dH_2O and diluted to 0.2 OD_{600}/ml (5 ml) with liquid Low Iron Media for growth at 24°C for 72 hr. Three microliters of India ink were added to 97 µl of cell suspension. The images were taken at 1000X magnification and photographed. Scale bar = 5 µm.

(B) *C. gattii* strain WM276 produced a larger capsule radius compared with the *C. gattii* R265 and R272, and the *C. neoformans* strain H99. Capsule radius was measured from ~50 cells for each strain and plotted with Prism 5.03. *** $P < 0.0001$ (unpaired *t* test).

(C) Divergent roles of calcineurin on capsule production. Capsule radius of wild-type and calcineurin mutants was measured as described above and plotted with Prism 5.03. The *P* values are indicated (Dunnett's multiple comparison test for comparing *C. gattii* wild-type and two independent mutants, while an unpaired *t* test was used to compare *C. neoformans* wild-type and the calcineurin mutant strain KK1).

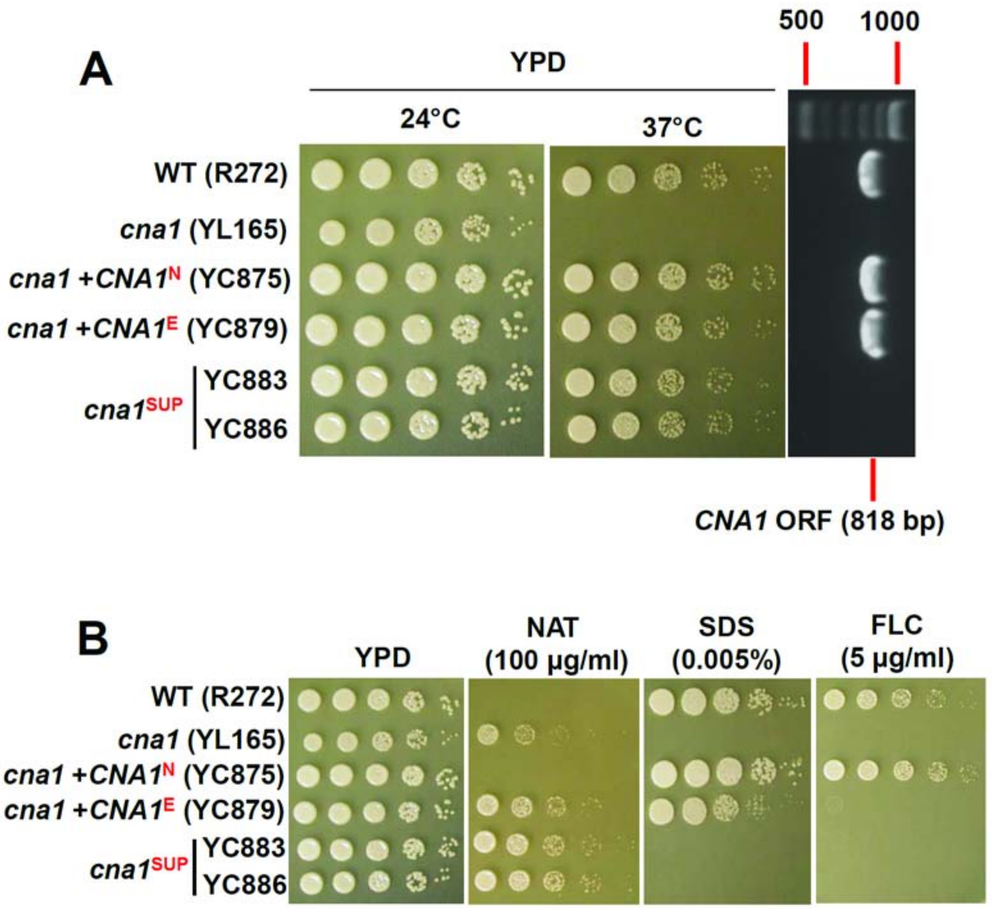

Figure S7 Complementation of R272 *cna1* mutant and isolation of suppressor mutations at 38°C.
(A) In the *CNA1* complemented strains YC875 (native-locus integration) and YC879 (ectopic integration), and two independently derived suppressor mutants YC883 and YC886 the ability of the *cna1* mutant YL165 to grow at 37°C was complemented or suppressed and growth was restored at 37°C. An 818 bp region of the *CNA1* ORF could be PCR amplified with primers JC203/JC204 (right panel) from complemented strains YC875 and YC879, but not from the suppressor mutant strains YC883 and YC886. The red superscripts N, E, and SUP represent 'Native', 'Ectopic', and 'Suppressor' respectively.
(B) *CNA1* native-locus complemented strain YC875 was fully rescued for the *cna1* mutant phenotype in response to the cell wall integrity-damaging agent SDS or fluconazole, while the *CNA1* etopically complemented strain YC879 was only partially rescued for phenotypes on SDS, but not fluconazole. The suppressor mutants YC883 and YC886 were not rescued for phenotypes on SDS or fluconazole.

File S1

Supplemental Results

Calcineurin is not required for melanin production

Melanins, widely distributed in the living world, are high molecular weight pigments formed by oxidative polymerization of phenolic compounds and are usually dark brown or black in color (Jacobson, 2000). Melanin synthesis has been associated with virulence in several pathogenic fungi, including *C. neoformans* (Casadevall *et al.*, 2000). *C. neoformans* also produces melanin *in vivo* during human or rodent infections (Nosanchuk *et al.*, 2000; Rosas *et al.*, 2000), and strains that are unable to synthesize melanin have attenuated virulence in animal infection models (Casadevall *et al.*, 2000). This correlation between melanin and virulence is most likely due to the ability of melanized cells to avoid being phagocytosed by macrophages as well as their ability to down-regulate the T-cell-mediated immune response (Rosas *et al.*, 2000). *C. neoformans* requires a phenolic substrate in order to produce melanin *in vitro,* and it is predicted that it is able to synthesize the product *in vivo* by taking advantage of host catecholamines such as DOPA and norepenephrine (Garcia-Rivera *et al.*, 2005). Laccase, encoded by the *LAC1* gene in *C. neoformans,* is the enzyme that is required to convert phenolic substances to polymerized melanin (Casadevall *et al.*, 2000). Here we investigated the roles of calcineurin in melanin production in *C. gattii* and *C. neoformans*. We found that calcineurin is not required for melanin production in these two species, as demonstrated by similar melanin production levels on Niger seed and L-DOPA agar plates between wild-type and calcineurin mutants (Figure S3).

***C. gattii* calcineurin plays minor roles in controlling capsule production**

Capsule production is a key virulence determinant for pathogenic fungi, and its synthesis is regulated by iron levels (Lian *et al.*, 2005). The capsule is thought to either protect a cell from being engulfed by phagocytes or to allow the cell to survive inside of a phagocyte. The capsule may also suppress inflammatory cells and influence antibody production (Lian *et al.*, 2005). Here, we investigated how capsule production in different *C. gattii* and *C. neoformans* isolates is affected by a low iron environment. We found that the environmental *C. gattii* WM276 isolate produced larger capsules under low iron conditions compared with the clinical *C. gattii* isolates R265 and R272, and *C. neoformans* H99 (Figures S6A and S6B; $P < 0.0001$; unpaired *t* test). However, the roles of *C. neoformans* calcineurin in capsule production remain elusive. We found that calcineurin mutants from *C. gattii* R265 and R272 VGII strains exhibited a larger capsule when compared with their wild-type (Figure S6C, $P < 0.05$; ANOVA and Dunnett's multiple comparison test), while calcineurin mutants from *C. gattii* WM276 and *C. neoformans* H99 exhibited similar capsule size compared with their wild-type counterpart (Figure S6C; $P > 0.05$).

LITERATURE CITED

Casadevall, A., Rosas, A.L., and Nosanchuk, J.D. (2000) Melanin and virulence in *Cryptococcus neoformans*. *Curr Opin Microbiol* **3**: 354-358.

Garcia-Rivera, J., Eisenman, H.C., Nosanchuk, J.D., Aisen, P., Zaragoza, O., Moadel, T., Dadachova, E., and Casadevall, A. (2005) Comparative analysis of *Cryptococcus neoformans* acid-resistant particles generated from pigmented cells grown in different laccase substrates. *Fungal Genet Biol* **42**: 989-998.

Jacobson, E.S. (2000) Pathogenic roles for fungal melanins. *Clin Microbiol Rev* **13**: 708-717.

Lian, T., Simmer, M.I., D'Souza, C.A., Steen, B.R., Zuyderduyn, S.D., Jones, S.J., Marra, M.A., and Kronstad, J.W. (2005) Iron-regulated transcription and capsule formation in the fungal pathogen *Cryptococcus neoformans*. *Mol Microbiol* **55**: 1452-1472.

Nosanchuk, J.D., Rosas, A.L., Lee, S.C., and Casadevall, A. (2000) Melanisation of *Cryptococcus neoformans* in human brain tissue. *Lancet* **355**: 2049-2050.

Rosas, A.L., Nosanchuk, J.D., Feldmesser, M., Cox, G.M., McDade, H.C., and Casadevall, A. (2000) Synthesis of polymerized melanin by *Cryptococcus neoformans* in infected rodents. *Infect Immun* **68**: 2845-2853.

Table S1 PCR primers used in this study.

Primer	Use	Sequence (5’ → 3’)
JC65	*NAT* marker (M13 Forward)	GTAAAACGACGGCCAGT
JC66	*NAT* marker (M13 Reverse)	GGAAACAGCTATGACCATG
JC174	R265 & R272 5’ NCR of *CNA1*	TCTTGGGATTAGCCTCTCCCT
JC175	R265 & R272 5’ NCR of *CNA1*	ACTGGCCGTCGTTTTACGGAAGTTGACTGATTGGTGGT
JC176	R265 & R272 3’ NCR of *CNA1*	CATGGTCATAGCTGTTTCCAGTTTCGAACGATGGAATCG
JC177	R265 & R272 3’ NCR of *CNA1*	TTGGCTGACAAACCCGCTA
JC178	R265 & R272 Overlap PCR	GGAAGGCCAAAGGATTTACA
JC179	R265 & R272 Overlap PCR	AAGGTAAGACTCCAGGCGAA
JC203	R265 & R272 *CNA1 ORF*	TCAGAACCAAACTTGCTGGA
JC204	R265 & R272 *CNA1 ORF*	TCCAAGAACTGACATGCAGCA
JC207	WM276 5’ NCR of *CNA1*	TCTTGGGATTAGCCTCTCCCT
JC208	WM276 5’ NCR of *CNA1*	ACTGGCCGTCGTTTTACAAGTTGGCTGACTAGTGGTGG
JC209	WM276 3’ NCR of *CNA1*	CATGGTCATAGCTGTTTCCTGATCGAATCGTTTGAACGAC
JC210	WM276 3’ NCR of *CNA1*	TGCCAAAAAAACTTGACGTCTG
JC211	WM276 overlap PCR	AGGAAGGCCAAAGGATTTACA
JC212	WM276 overlap PCR	TACCCTTACATCTTTGTACG
JOHE23397	WM276 *CNA1* ORF	GGACGTGTTCACCTGGAGTT
JOHE23398	WM276 *CNA1* ORF	AGCAAGGCTTAA TGGCAGAA
