# Supplementary material for: Calcineurin Governs Thermotolerance and Virulence of Cryptococcus gattii
Source: G3 (Bethesda). 2013 Mar 1;3(3):527–39. doi: 10.1534/g3.112.004242 (PMC3583459; doi:10.1534/g3.112.004242)
Supplement: Supporting Information [file supp_3.3.527_FigureS1.pdf]

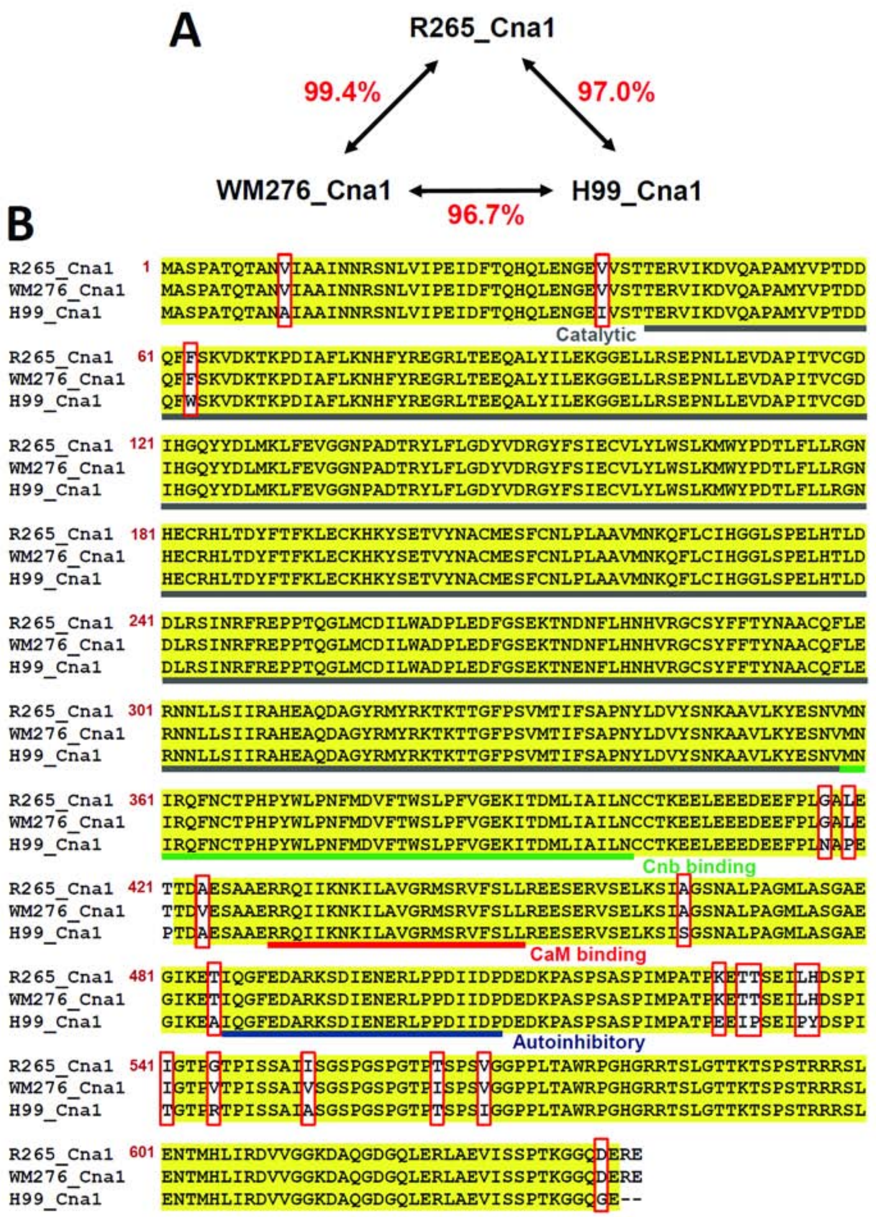

Figure S1 Amino acid identity and pairwise alignments of calcineurin catalytic subunit (Cna1) from *C. gattii* R265, *C. gattii* WM276, and *C. neoformans* H99. Amino acid identity and multiple sequence alignments are depicted using ClustalW software (http://npsa-pbil.ibcp.fr/cgi-bin/npsa_automat.pl?page=npsa_clustalw.html). **(A)** The % identity shared between the full-length proteins is shown in red. **(B)** The conserved and divergent amino acids are indicated with yellow shading and red rectangle, respectively. The catalytic domain, calcineurin regulatory subunit (Cnb) binding, calmodulin (CaM) binding, and autoinhibitory domains are indicated with gray, green, red, and blue underlining, respectively. The amino acids of the *C. gattii* R265 Cna1 protein are numbered.
