# Supplementary material for: Calcineurin Governs Thermotolerance and Virulence of Cryptococcus gattii
Source: G3 (Bethesda). 2013 Mar 1;3(3):527–39. doi: 10.1534/g3.112.004242 (PMC3583459; doi:10.1534/g3.112.004242)
Supplement: Supporting Information [file supp_3.3.527_FigureS2.pdf]

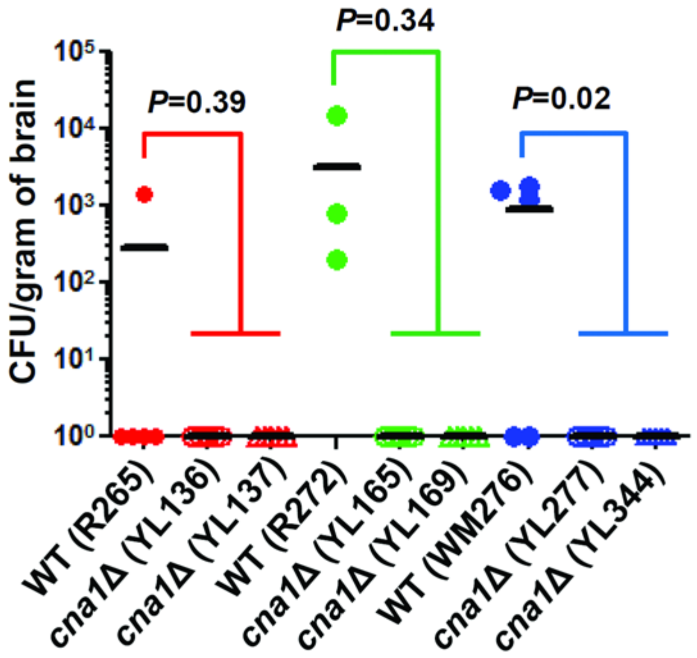

Figure S2 The fungal burden of *C. gattii* wild-type and calcineurin mutants in the brain. The fungal burden in the brain was determined at 14 days post-infection ($5x10^4$ cells per mouse). Five mice per strain were used. *P* values were determined by ANOVA and Dunnett's multiple comparison tests.
