# Supplementary material for: Calcineurin Governs Thermotolerance and Virulence of Cryptococcus gattii
Source: G3 (Bethesda). 2013 Mar 1;3(3):527–39. doi: 10.1534/g3.112.004242 (PMC3583459; doi:10.1534/g3.112.004242)
Supplement: Supporting Information [file supp_3.3.527_FigureS3.pdf]

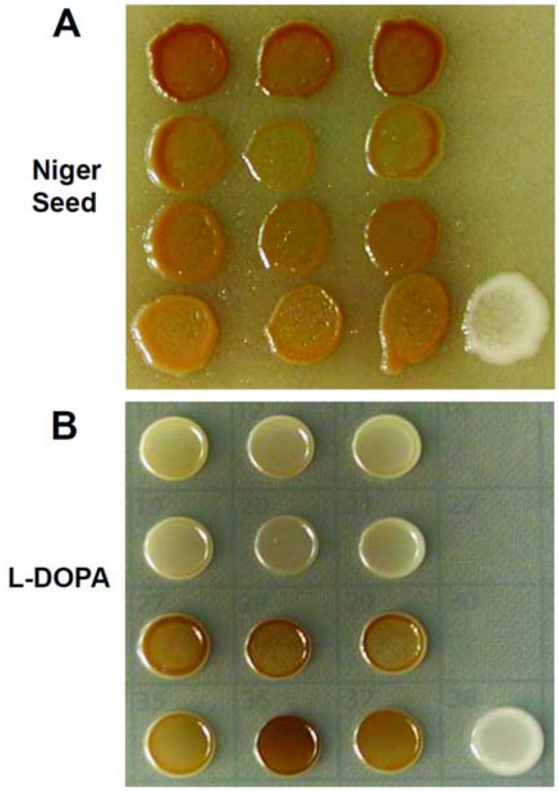

C

R265	*cna1*Δ (YL136)	*cna1*Δ (YL137)	
R272	*cna1*Δ (YL165)	*cna1*Δ (YL169)	
WM276	*cna1*Δ (YL277)	*cna1*Δ (YL344)	
H99	*cna1*Δ (KK1)	*cna1*Δ + *CNA1* (KK5)	*lac1*

Figure S3 Calcineurin is not required for melanin production in *C. gattii* and *C. neoformans*. Cells were grown overnight at 24°C, washed twice with dH_2O, diluted to 1 OD_{600}/ml, and 3 µl of cell suspension was plated on Niger seed **(A)** and L-DOPA **(B)** agar medium and incubated for 72 hr at 24°C. Strains analyzed are indicated in panel (**C)**.
