# Supplementary material for: Calcineurin Governs Thermotolerance and Virulence of Cryptococcus gattii
Source: G3 (Bethesda). 2013 Mar 1;3(3):527–39. doi: 10.1534/g3.112.004242 (PMC3583459; doi:10.1534/g3.112.004242)
Supplement: Supporting Information [file supp_3.3.527_FigureS4.pdf]

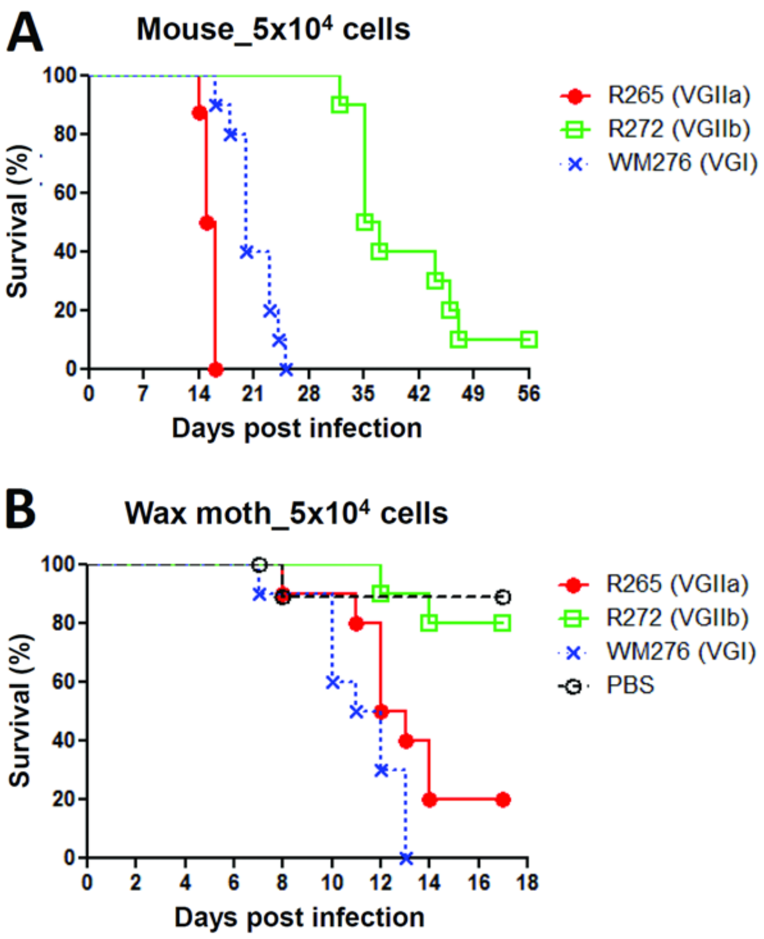

Figure S4 Comparison of *C. gattii* wild-type virulence in both murine inhalation and wax moth models. The data of *C. gattii* wild-type virulence in the murine inhalation (A) and wax moth models (B) were extracted from Figure 4 and Figure S5. Each mouse or wax moth received $5x10^4$ *C. gattii* yeast cells.
