# Supplementary material for: Calcineurin Governs Thermotolerance and Virulence of Cryptococcus gattii
Source: G3 (Bethesda). 2013 Mar 1;3(3):527–39. doi: 10.1534/g3.112.004242 (PMC3583459; doi:10.1534/g3.112.004242)
Supplement: Supporting Information [file supp_3.3.527_FigureS5.pdf]

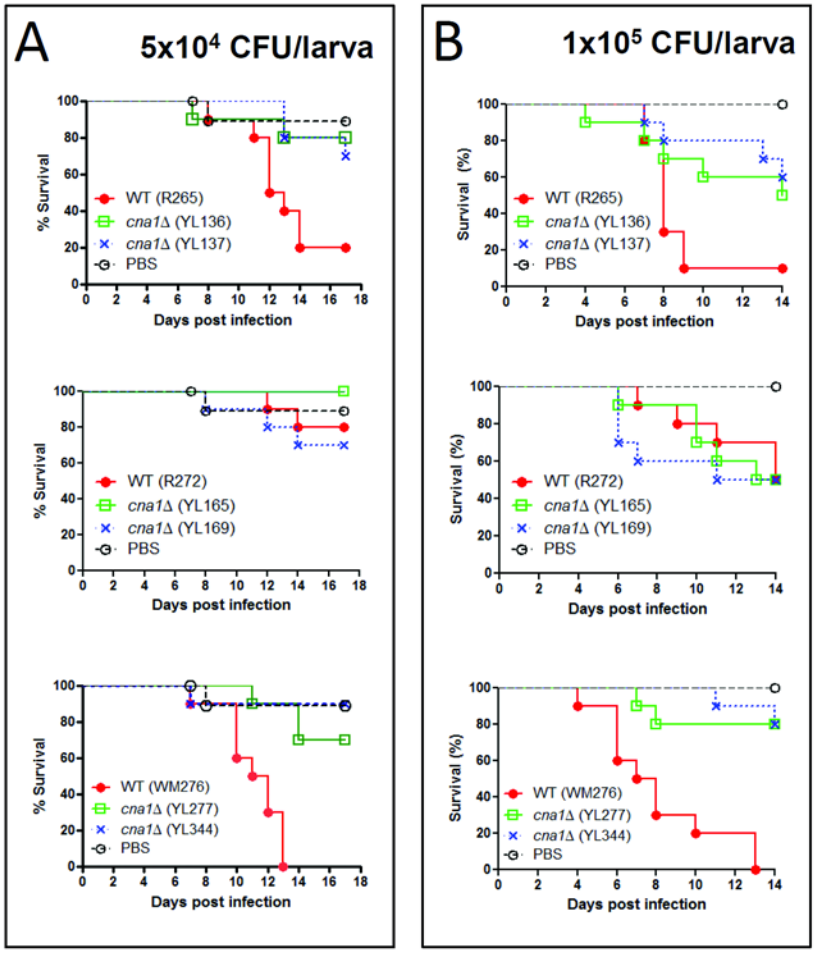

Figure S5 Roles of *C. gattii* calcineurin in the wax moth model. Survival of *Galleria mellonella* after injection of 5x10^4 **(A)** or 10^5 **(B)** CFU/larva of *C. gattii* wild-type or calcineurin mutants (10 moths per isolate).
