# Supplementary material for: Calcineurin Governs Thermotolerance and Virulence of Cryptococcus gattii
Source: G3 (Bethesda). 2013 Mar 1;3(3):527–39. doi: 10.1534/g3.112.004242 (PMC3583459; doi:10.1534/g3.112.004242)
Supplement: Supporting Information [file supp_3.3.527_FigureS6.pdf]

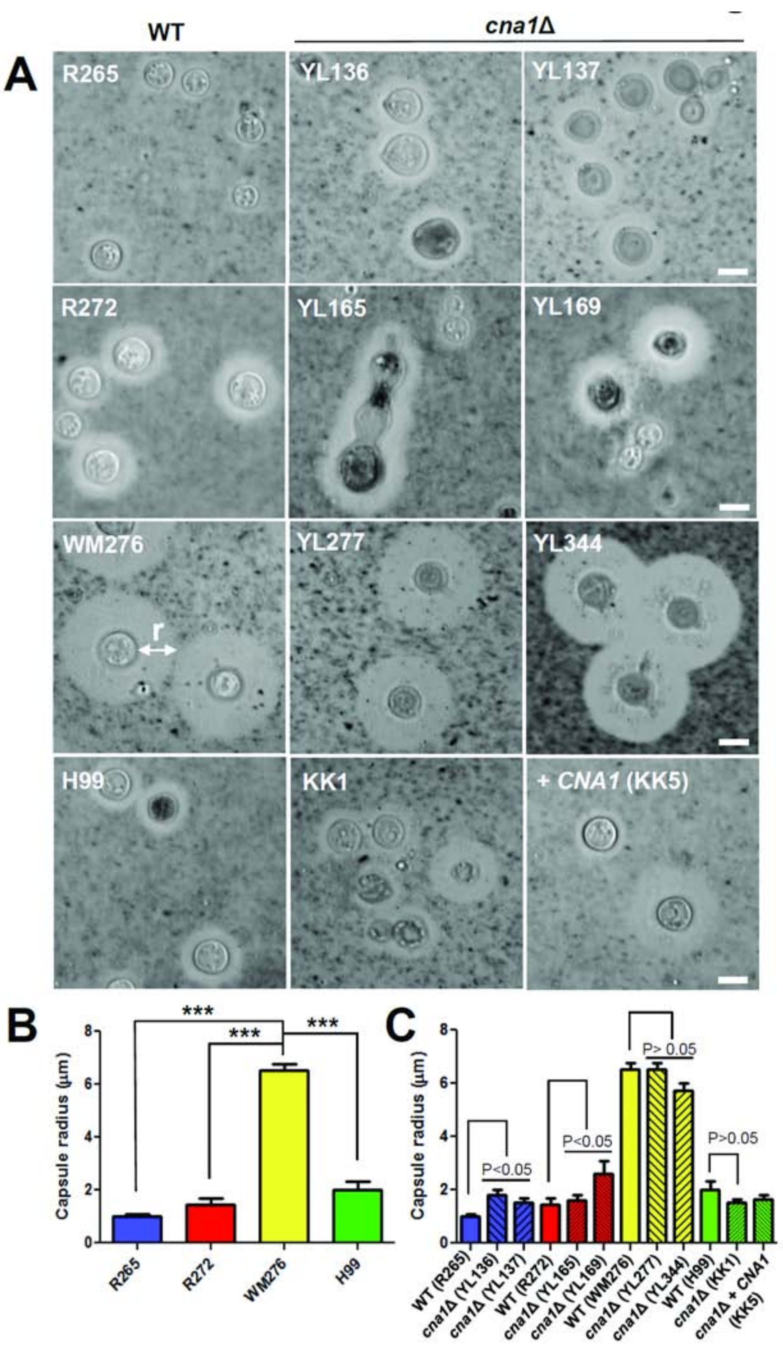

Figure S6 Calcineurin plays minor roles in capsule production.

(A) Capsule radius (labeled with r) of wild-type and calcineurin mutants was determined with India ink staining. Cells were grown overnight at 24°C in YPD media. Cells were washed twice with dH_2O and diluted to 0.2 OD_{600}/ml (5 ml) with liquid Low Iron Media for growth at 24°C for 72 hr. Three microliters of India ink were added to 97 µl of cell suspension. The images were taken at 1000X magnification and photographed. Scale bar = 5 µm.

(B) *C. gattii* strain WM276 produced a larger capsule radius compared with the *C. gattii* R265 and R272, and the *C. neoformans* strain H99. Capsule radius was measured from ~50 cells for each strain and plotted with Prism 5.03. *** $P < 0.0001$ (unpaired *t* test).

(C) Divergent roles of calcineurin on capsule production. Capsule radius of wild-type and calcineurin mutants was measured as described above and plotted with Prism 5.03. The *P* values are indicated (Dunnett's multiple comparison test for comparing *C. gattii* wild-type and two independent mutants, while an unpaired *t* test was used to compare *C. neoformans* wild-type and the calcineurin mutant strain KK1).
