# Supplementary material for: Calcineurin Governs Thermotolerance and Virulence of Cryptococcus gattii
Source: G3 (Bethesda). 2013 Mar 1;3(3):527–39. doi: 10.1534/g3.112.004242 (PMC3583459; doi:10.1534/g3.112.004242)
Supplement: Supporting Information [file supp_3.3.527_FigureS7.pdf]

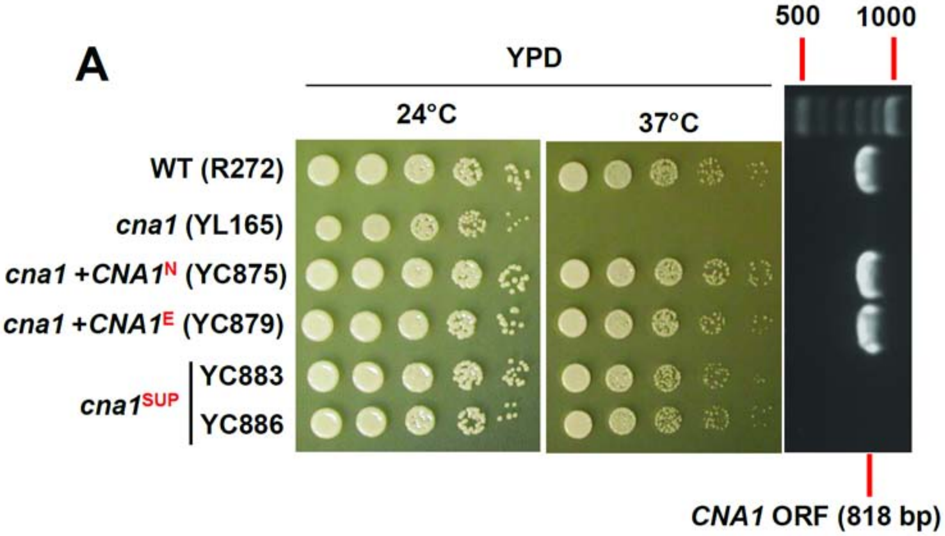


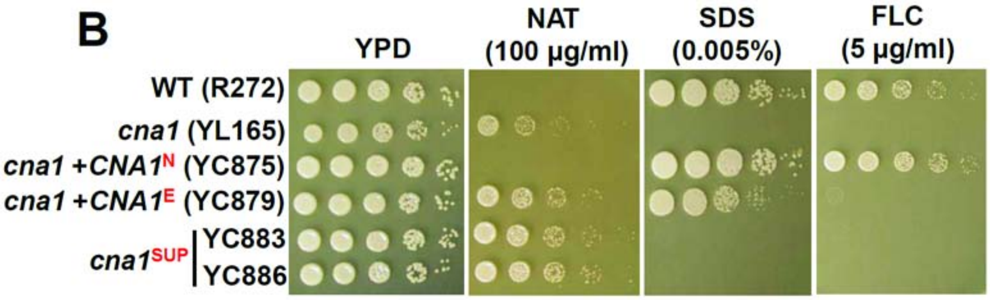


**Figure S7 Complementation of R272 *cna1* mutant and isolation of suppressor mutations at 38°C.**
**(A)** In the *CNA1* complemented strains YC875 (native-locus integration) and YC879 (ectopic integration), and two independently derived suppressor mutants YC883 and YC886 the ability of the *cna1* mutant YL165 to grow at 37°C was complemented or suppressed and growth was restored at 37°C. An 818 bp region of the *CNA1* ORF could be PCR amplified with primers JC203/JC204 (right panel) from complemented strains YC875 and YC879, but not from the suppressor mutant strains YC883 and YC886. The red superscripts N, E, and SUP represent 'Native', 'Ectopic', and 'Suppressor' respectively.
**(B)** *CNA1* native-locus complemented strain YC875 was fully rescued for the *cna1* mutant phenotype in response to the cell wall integrity-damaging agent SDS or fluconazole, while the *CNA1* etopically complemented strain YC879 was only partially rescued for phenotypes on SDS, but not fluconazole. The suppressor mutants YC883 and YC886 were not rescued for phenotypes on SDS or fluconazole.
