# Supplementary material for: Calcineurin Governs Thermotolerance and Virulence of Cryptococcus gattii
Source: G3 (Bethesda). 2013 Mar 1;3(3):527–39. doi: 10.1534/g3.112.004242 (PMC3583459; doi:10.1534/g3.112.004242)
Supplement: Supporting Information [file supp_3.3.527_FileS1.pdf]

File S1

Supplemental Results

Calcineurin is not required for melanin production

Melanins, widely distributed in the living world, are high molecular weight pigments formed by oxidative polymerization of phenolic compounds and are usually dark brown or black in color (Jacobson, 2000). Melanin synthesis has been associated with virulence in several pathogenic fungi, including *C. neoformans* (Casadevall *et al.*, 2000). *C. neoformans* also produces melanin *in vivo* during human or rodent infections (Nosanchuk *et al.*, 2000; Rosas *et al.*, 2000), and strains that are unable to synthesize melanin have attenuated virulence in animal infection models (Casadevall *et al.*, 2000). This correlation between melanin and virulence is most likely due to the ability of melanized cells to avoid being phagocytosed by macrophages as well as their ability to down-regulate the T-cell-mediated immune response (Rosas *et al.*, 2000). *C. neoformans* requires a phenolic substrate in order to produce melanin *in vitro,* and it is predicted that it is able to synthesize the product *in vivo* by taking advantage of host catecholamines such as DOPA and norepenephrine (Garcia-Rivera *et al.*, 2005). Laccase, encoded by the *LAC1* gene in *C. neoformans,* is the enzyme that is required to convert phenolic substances to polymerized melanin (Casadevall *et al.*, 2000). Here we investigated the roles of calcineurin in melanin production in *C. gattii* and *C. neoformans*. We found that calcineurin is not required for melanin production in these two species, as demonstrated by similar melanin production levels on Niger seed and L-DOPA agar plates between wild-type and calcineurin mutants (Figure S3).

***C. gattii* calcineurin plays minor roles in controlling capsule production**

Capsule production is a key virulence determinant for pathogenic fungi, and its synthesis is regulated by iron levels (Lian *et al.*, 2005). The capsule is thought to either protect a cell from being engulfed by phagocytes or to allow the cell to survive inside of a phagocyte. The capsule may also suppress inflammatory cells and influence antibody production (Lian *et al.*, 2005). Here, we investigated how capsule production in different *C. gattii* and *C. neoformans* isolates is affected by a low iron environment. We found that the environmental *C. gattii* WM276 isolate produced larger capsules under low iron conditions compared with the clinical *C. gattii* isolates R265 and R272, and *C. neoformans* H99 (Figures S6A and S6B; $P < 0.0001$; unpaired *t* test). However, the roles of *C. neoformans* calcineurin in capsule production remain elusive. We found that calcineurin mutants from *C. gattii* R265 and R272 VGII strains exhibited a larger capsule when compared with their wild-type (Figure S6C, $P < 0.05$; ANOVA and Dunnett's multiple comparison test), while calcineurin mutants from *C. gattii* WM276 and *C. neoformans* H99 exhibited similar capsule size compared with their wild-type counterpart (Figure S6C; $P > 0.05$).

LITERATURE CITED

Casadevall, A., Rosas, A.L., and Nosanchuk, J.D. (2000) Melanin and virulence in *Cryptococcus neoformans*. *Curr Opin Microbiol* **3**: 354-358.

Garcia-Rivera, J., Eisenman, H.C., Nosanchuk, J.D., Aisen, P., Zaragoza, O., Moadel, T., Dadachova, E., and Casadevall, A. (2005) Comparative analysis of *Cryptococcus neoformans* acid-resistant particles generated from pigmented cells grown in different laccase substrates. *Fungal Genet Biol* **42**: 989-998.

Jacobson, E.S. (2000) Pathogenic roles for fungal melanins. *Clin Microbiol Rev* **13**: 708-717.

Lian, T., Simmer, M.I., D'Souza, C.A., Steen, B.R., Zuyderduyn, S.D., Jones, S.J., Marra, M.A., and Kronstad, J.W. (2005) Iron-regulated transcription and capsule formation in the fungal pathogen *Cryptococcus neoformans*. *Mol Microbiol* **55**: 1452-1472.

Nosanchuk, J.D., Rosas, A.L., Lee, S.C., and Casadevall, A. (2000) Melanisation of *Cryptococcus neoformans* in human brain tissue. *Lancet* **355**: 2049-2050.

Rosas, A.L., Nosanchuk, J.D., Feldmesser, M., Cox, G.M., McDade, H.C., and Casadevall, A. (2000) Synthesis of polymerized melanin by *Cryptococcus neoformans* in infected rodents. *Infect Immun* **68**: 2845-2853.
