# Supplementary material for: Calcineurin Governs Thermotolerance and Virulence of Cryptococcus gattii
Source: G3 (Bethesda). 2013 Mar 1;3(3):527–39. doi: 10.1534/g3.112.004242 (PMC3583459; doi:10.1534/g3.112.004242)
Supplement: Supporting Information [file supp_3.3.527_TableS1.pdf]

Table S1 PCR primers used in this study.

Primer	Use	Sequence (5’ → 3’)
JC65	*NAT* marker (M13 Forward)	GTAAAACGACGGCCAGT
JC66	*NAT* marker (M13 Reverse)	GGAAACAGCTATGACCATG
JC174	R265 & R272 5’ NCR of *CNA1*	TCTTGGGATTAGCCTCTCCCT
JC175	R265 & R272 5’ NCR of *CNA1*	ACTGGCCGTCGTTTTACGGAAGTTGACTGATTGGTGGT
JC176	R265 & R272 3’ NCR of *CNA1*	CATGGTCATAGCTGTTTCCAGTTTCGAACGATGGAATCG
JC177	R265 & R272 3’ NCR of *CNA1*	TTGGCTGACAAACCCGCTA
JC178	R265 & R272 Overlap PCR	GGAAGGCCAAAGGATTTACA
JC179	R265 & R272 Overlap PCR	AAGGTAAGACTCCAGGCGAA
JC203	R265 & R272 *CNA1 ORF*	TCAGAACCAAACTTGCTGGA
JC204	R265 & R272 *CNA1 ORF*	TCCAAGAACTGACATGCAGCA
JC207	WM276 5’ NCR of *CNA1*	TCTTGGGATTAGCCTCTCCCT
JC208	WM276 5’ NCR of *CNA1*	ACTGGCCGTCGTTTTACAAGTTGGCTGACTAGTGGTGG
JC209	WM276 3’ NCR of *CNA1*	CATGGTCATAGCTGTTTCCTGATCGAATCGTTTGAACGAC
JC210	WM276 3’ NCR of *CNA1*	TGCCAAAAAACTTGACGTCTG
JC211	WM276 overlap PCR	AGGAAGGCCAAAGGATTTACA
JC212	WM276 overlap PCR	TACCCTTACATCTTTGTACG
JOHE23397	WM276 *CNA1* ORF	GGACGTGTTCACCTGGAGTT
JOHE23398	WM276 *CNA1* ORF	AGCAAGGCTTAA TGGCAGAA
